# Targeting Amastigote and Trypomastigote Phases: Multi‐Epitope Vaccine Strategy Against *Trypanosoma cruzi*

**DOI:** 10.1002/bab.70185

**Published:** 2026-05-29

**Authors:** Maria Karolaynne da Silva, Caio Patrício de Souza Sena, Davi Serradella Vieira, Magdi E. A. Zaki, Leow Chiuan Yee, Shahina Akter, Umberto Laino Fulco, Jonas Ivan Nobre Oliveira, João Firmino Rodrigues‐Neto

**Affiliations:** ^1^ Department of Biophysics and Pharmacology, Bioscience Center Federal University of Rio Grande Do Norte Natal Brazil; ^2^ Chemistry Institute Federal University of Rio Grande Do Norte Natal Brazil; ^3^ Department of Chemistry, College of Science Imam Mohammad Ibn Saud Islamic University (IMSIU) Riyadh Saudi Arabia; ^4^ Institute For Research in Molecular Medicine Universiti Sains Malaysia Kelantan Malaysia; ^5^ Bangladesh Council of Scientific & Industrial Research (BCSIR) Dhaka Bangladesh; ^6^ Department of Clinical and Toxicological Analysis Federal University of Rio Grande Do Norte Natal Brazil

**Keywords:** Chagas disease, immunoinformatics, multi‐epitope vaccine, *Trypanosoma cruzi*

## Abstract

Effective vaccines against *Trypanosoma cruzi*, the causative agent of Chagas disease, are urgently needed. Here, we report the design and in silico validation of a novel multiepitope vaccine construct targeting the key surface proteins ASP‐2 and gp82. Using a comprehensive immunoinformatics pipeline, we identified and selected 38 potent T‐cell (CTL/HTL) and B‐cell epitopes, ensuring high antigenicity, immunogenicity, and safety. The final chimeric protein, integrated with adjuvants and stabilizing linkers, demonstrated favorable physicochemical properties, high solubility, and was predicted to be non‐allergenic and non‐toxic, with a significant population coverage of approximately 62% in Latin America. Structural modeling and refinement confirmed a stable tertiary structure. Crucially, molecular docking predicted high‐affinity interactions with the immune receptors TLR2 and TLR4 (docking scores: −1360.4 and −1278.7, respectively). The stability and flexibility of these vaccine–receptor complexes were further validated by 300 ns molecular dynamics simulations. Finally, codon optimization and in silico cloning projected high expression potential in an *Escherichia coli* system. Immune simulations predicted robust responses: Innate (elevated cytokines, dendritic cell activation), humoral (IgG/IgM production), and cellular (CD4+/CD8+/memory T‐cell activation) across simulated doses. These findings strongly support the potential of this vaccine candidate and provide a solid foundation for its further preclinical development against *T. cruzi*.

## 1 Introduction

Chagas disease, caused by the protozoan parasite *Trypanosoma cruzi*, remains a significant neglected tropical disease, affecting millions primarily in Latin America and posing a growing global health concern due to human migration [1]. With treatment limited to the acute phase and no effective vaccine available, the development of a prophylactic immunization strategy is a critical priority for disease control [2], which necessitates the exploration of diverse antigens for vaccine development.

The quest for a vaccine against Chagas disease has spanned decades, exploring various platforms including attenuated parasites, DNA vaccines, viral vectors, and recombinant subunit proteins [1, 3, 4, 5]. While some candidates have shown promise in preclinical models by reducing parasite load and inflammation, significant challenges persist. The complex life cycle of *T. cruzi*, alternating between extracellular trypomastigotes and intracellular amastigotes, requires the induction of a multifaceted immune response, encompassing both neutralizing antibodies and potent CD4+ and CD8+ T‐cell effectors to achieve sterile immunity [2, 6, 7, 8].

Among the numerous antigens investigated, Amastigote Surface Protein 2 (ASP‐2) has emerged as a leading vaccine candidate. Primarily expressed on the surface of intracellular amastigotes, ASP‐2 is crucial for parasite survival. More importantly, preclinical studies have extensively validated its immunogenicity, demonstrating that immunization with DNA or recombinant ASP‐2 induces robust, protective CD8+ T‐cell responses that are essential for controlling parasite burden and myocarditis in murine models [9, 10, 11]. This evidence substantiates ASP‐2's role as a powerful target for inducing a cellular response against the chronic stage of the infection.

Complementing this, the surface glycoprotein gp82, expressed by metacyclic trypomastigotes, plays a pivotal role in the initial host cell invasion process [12]. The gp82 protein mediates parasite attachment and entry into diverse cell types, making it a key target for neutralizing antibodies. Studies have shown that *T. cruzi* metacyclic forms deficient in gp82 fail to promote efficient infection, thus antibodies targeting gp82 can effectively block cell invasion, suggesting that its inclusion in a vaccine formulation could prevent the initial establishment and spread of infection [13, 14, 15, 16].

Given the distinct roles and immune responses elicited by these antigens, a strategy that simultaneously targets different life stages of the parasite offers a powerful and rational approach. We hypothesize that a multi‐stage vaccine combining the cellular immunity‐inducing potential of ASP‐2 with the humoral immunity‐inducing capacity of gp82 could provide more comprehensive and effective protection than single‐antigen approaches. This dual‐target design aims to equip the host immune system to fight both the invasive trypomastigote and the established amastigote forms of the parasite.

In this context, immunoinformatics provides an accelerated, cost‐effective, and systematic framework for designing and optimizing complex chimeric vaccines. By predicting and selecting the most potent T‐cell and B‐cell epitopes from target antigens, it is possible to construct a single immunogen with enhanced safety, stability, and immunogenic potential [17, 18]. Therefore, this study aims to design and perform an extensive in silico validation of a novel multi‐epitope vaccine against *T. cruzi*, integrating highly immunogenic epitopes from ASP‐2 and gp82, to create a promising candidate for future preclinical evaluation.

## 2 Methods

### 2.1 Acquisition of *T. cruzi* Protein Sequences

To establish reference sequences for the *T. cruzi* proteins gp82 and ASP‐2, a consensus sequence was generated for each. First, all available protein sequences for gp82 (*n* = 36) and ASP‐2 (*n* = 13) were retrieved from the NCBI Protein Database (https://www.ncbi.nlm.nih.gov/protein) in FASTA format. The sequences for each protein set were then separately aligned using the MAFFT server (https://mafft.cbrc.jp/alignment/server/), which employs fast Fourier transform (FFT) algorithms to rapidly identify homologous regions and construct multiple sequence alignments. Finally, the resulting alignments were imported into JALVIEW (version 2.11.1.4) [19] to derive the definitive consensus sequence for each protein used in this study.

### 2.2 Prediction of T‐Cell Epitopes

#### 2.2.1 Cytotoxic T‐Cell Lymphocytes

The prediction of MHC class I restricted (Cytotoxic T‐Cell Lymphocyte [CTL]) epitopes involved the utilization of two web servers, namely NetMHCpan‐4.1 and NetCTL. NetMHCpan4.1 (https://services.healthtech.dtu.dk/service.php?NetMHCpan‐4.1) predicts binding of peptides to any MHC molecule of known sequence using artificial neural networks (ANNs). The method is trained on a combination of over 850,000 quantitative Binding Affinity (BA) and Mass‐Spectrometry Eluted Ligands (EL) peptides [20]. To validate the epitopes identified by NetMHCpan4.1, the NetCTL server (https://services.healthtech.dtu.dk/ services/NetCTL‐1.2/) was employed. NetCTL integrates an ANN architecture to predict MHC class I binding and proteasomal C‐terminal cleavage, alongside a weight matrix algorithm to estimate TAP transport efficiency. Cutoffs for C‐terminal cleavage, TAP transport efficiency, and CTL epitope identification were set at 0.75, 0.15, and 0.05, respectively, ensuring the selection of high‐quality potential epitopes for further analysis [21].

#### 2.2.2 Helper T‐Lymphocytes

In the estimation of Helper T‐Lymphocyte (HTL) epitopes binding to the HLA‐DQ, HLA‐DP, and HLA‐DR alleles, two methods utilizing ANNs were employed. The first method utilized the NetMHCII web server (https://services.healthtech. dtu.dk/services/NetMHCII‐2.3/), which leverages a comprehensive dataset consisting of over 100,000 quantitative peptide binding measurements for accurate prediction [22]. To validate the predictions provided by NetMHCII, the protein sequences underwent analysis using the NetMHCIIpan server (https://services.healthtech. dtu.dk/services/NetMHCIIpan‐4.0/). This server also uses an ANN‐based algorithm trained on quantitative binding data and mass spectrometry EL, encompassing an extensive dataset comprising 8 mouse MHC‐ II molecules and 36 HLA‐DR, 27 HLA‐DQ, and 9 HLA‐DP molecules, thereby further enhancing the accuracy and reliability of HTL epitope estimation [23]. Our study specifically targeted the predominant HLA‐DR alleles prevalent in Latin American populations: HLA‐DRB1*01:01; HLA‐DRB1*03:01; HLA‐DRB1*04:01; HLA‐DRB1*07:01; HLA‐DRB1*08:02; HLA‐DRB1*09:01; HLA‐DRB1*11:01; HLA‐DRB1*13:02; HLA‐DRB1*14:02; HLA‐DRB1*15:01; and HLA‐DRB1*16:01.

### 2.3 Linear B Cell Epitopes

For prediction of linear B epitopes in ASP‐2 and gp82 proteins, the sequences were subjected to analysis using the online tool IEDB (Immune Epitope Database) (http://tools.immuneepitope.org/bcell/), employing the Kolaskar and Tongaonkar method [24]. This is a semi‐empirical algorithm that utilizes the physicochemical properties of amino acid residues and their occurrence frequencies in experimentally determined epitopes to calculate an antigenic propensity score. This method relies on the presence of amino acid residues in experimentally determined epitopes. The application of this method can predict antigenic determinants with approximately 75% accuracy [24]. To validate the obtained epitopes, the BCEpred web server (http://crdd.osdd.net/raghava/bcepred/) was employed. This web server is based on a plethora of physicochemical propensity scales utilizing amino acid properties, such as hydrophilicity and antigenicity, either individually or in combination. The antigenic propensity (Kolaskar) was selected with a threshold of 1.9. Results from both prediction tools were aligned simultaneously, and overlapping sequences were considered as B‐cell epitopes.

### 2.4 Evaluation of Antigenicity, Allergenicity, Immunogenicity, and Toxicity

To assess the antigenicity of the obtained consensus sequence, we employed the VaxiJen v2.0 web program (http://www.ddg‐pharmfac.net/vaxijen/VaxiJen/VaxiJen.html), setting the prediction accuracy threshold at 0.4 to optimize accuracy [25]. This server utilizes the auto‐cross covariance (ACC) transformation method, an alignment‐free approach that considers the physicochemical properties of amino acids, which are then classified using partial least squares (PLS) algorithms [25].

For evaluating the allergenicity, we utilized the AllerTop 2.0 server (http://www.ddg‐pharmfac.net/AllerTOP/). This tool also applies the ACC transformation method combined with a k‐nearest neighbor (kNN) machine learning algorithm to classify sequences based on an extensive training dataset. External validation tests have demonstrated the tool's capability to accurately detect 87% of allergens and 91% of non‐allergens [26]. This resource provided valuable insights into the allergenic potential of the epitopes, facilitating informed decisions across various applications.

To assess the immunogenicity of MHC‐I and MHC‐II epitopes, we employed the MHC class I (https://tools.iedb.org/immunogenicity/) and class II (http://tools.iedb.org/CD4episcore/) immunogenicity tools, respectively, available through the IEDB server, which rely on position‐specific scoring matrices (PSSM) and amino acid properties [27, 28].

Confirmation of the non‐toxicity of the selected epitopes was conducted using the ToxinPred web server (http://crdd.osdd.net/raghava/toxinpred/). This service employs support vector machine (SVM) machine learning techniques and quantitative matrix models to assess toxicity based on the epitopes’ physicochemical properties. The extensive database of ToxinPred comprises 1805 hazardous and 3593 non‐toxic peptides, ensuring reliable predictions [29]. Leveraging this tool provided crucial information on the safety profile of the selected epitopes, facilitating confident decision‐making across diverse applications.

### 2.5 Population Coverage Evaluation

Population coverage serves as a direct indicator of vaccine efficacy across different geographic regions by assessing the prevalence of human leukocyte antigen (HLA) alleles associated with the epitope of interest. To gauge the population coverage of the selected epitopes, we employed the IEDB Population Coverage tool (http://tools.iedb.org/population/), which uses an algorithm to calculate the fraction of individuals expected to respond to a given set of epitopes based on known HLA genotypic frequencies. This tool considers the distribution of MHC binding alleles, enabling the selection of peptides with diverse HLA binding specificities. In our investigation focusing on the Latin American population, population coverage was assessed by examining the proportion of individuals likely to respond to epitopes predicted to bind to both MHC‐I and MHC‐II alleles [30]. The most prevalent HLAs in Latin America were selected [31].

### 2.6 Pro‐Inflammatory Peptides

The T and B cell epitopes derived from ASP‐2 and gp82 proteins underwent analysis using the PIP‐EL server [32] to predict pro‐inflammatory responses. Within the PIP‐EL framework, a peptide was deemed pro‐inflammatory if it demonstrated the capacity to induce cytokines such as IL‐1alpha, IL‐1beta, TNF‐alpha, IL‐6, IL‐8, IL‐12, IL‐17, IL‐18, and IL‐23. This server integrates a comprehensive dataset comprising ten distinct models based on amino acid sequence, di‐peptide composition, distribution‐transition, and physicochemical properties. A peptide scoring above 0.45 is classified as an inducer of pro‐inflammatory cytokines.

### 2.7 Construction of Multi‐Epitope Vaccine Sequence

The CTL, HTL, and B‐cell epitopes obtained through the aforementioned immunoinformatic approaches were incorporated into the development of a vaccine sequence. To ensure appropriate linkage and presentation, specific linkers and adjuvants were utilized (Figure 1). Alanine‐rich linkers (AAY) were utilized for the fusion of CTL epitopes [33]. KK linkers were employed to connect linear B‐cell epitopes. Generally, the use of linkers enhances vaccine stability and enables each epitope to function independently post‐injection [34]. To enhance immunogenicity and elicit durable innate and adaptive immunity, the N‐terminus of the subunit vaccine was fused to two potent immunostimulatory adjuvants (β‐defensin‐4 and 50S ribosomal protein L7/L12) via the EAAAK linker. The selection of these adjuvants is crucial for stimulating TLR2 and TLR4, respectively.

**FIGURE 1:**
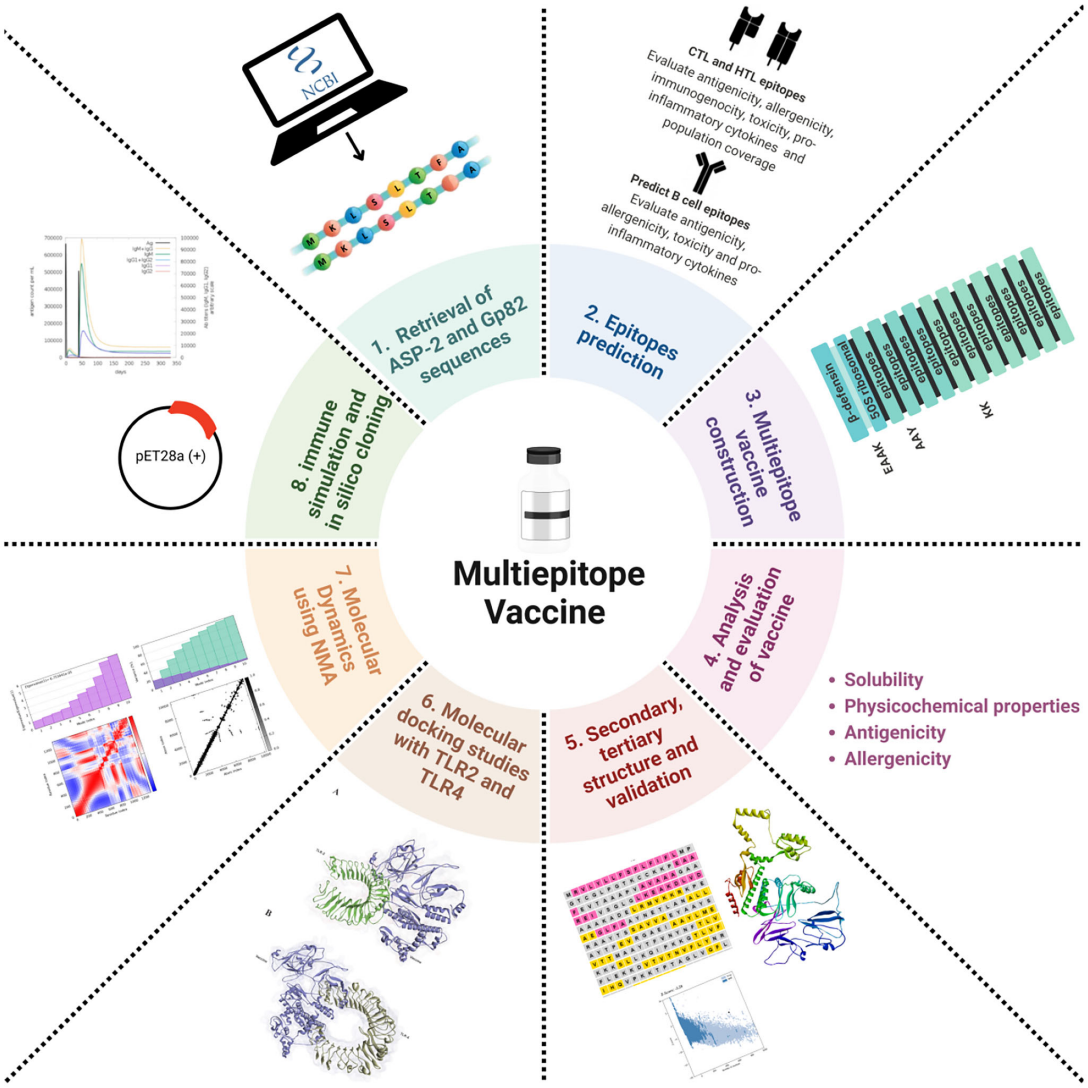
Schematic workflow for the in silico multiepitope vaccine design process.

### 2.8 Vaccine Physiochemical Properties, Antigenicity, Allergenicity, and Solubility Evaluation

We utilized the ProtParam tool available on the ExPASy server (https://web.expasy.org/cgi‐bin/protparam/protparam) to forecast the physiochemical properties of the engineered subunit vaccine. These properties encompassed the grand average of hydropathicity (GRAVY), amino acid count, amino acid composition, molecular weight (MW), theoretical isoelectric point (pI), half‐life, instability index, and aliphatic index [35]. In addition, a variety of web‐based tools were employed to anticipate the antigenicity (Vaxijen 2.0), allergenicity (Allertop 2.0), and solubility (SOLpro) of the modeled vaccine.

### 2.9 Vaccine Construct Secondary and Tertiary Structure Prediction

We utilized the PSIPRED server (http://bioinf.cs.ucl.ac.uk/psipred/) to generate the secondary structures of the vaccine [36]. PSIPRED evaluates the PSI‐BLAST output using two feed‐forward neural networks to provide simple and accurate 2D homology modeling [36]. The secondary structural patterns of the peptides were determined using SOPMA (https://npsa‐prabi.ibcp.fr/cgi‐bin/npsa_automat.pl?page=/NPSA/npsa_sopma.html) [37]. Subsequently, the Prabi server was employed to predict the proportion of secondary structural elements in the multi‐epitope vaccine (https://npsa‐prabi.ibcp.fr/cgi‐bin/npsa_automat.pl?page=/NPSA/npsa_gor4.html). GOR IV analysis achieved a prediction accuracy of 64.4% [38].

### 2.10 Prediction of Discontinuous (Conformational) B‐Cell Epitopes in the Vaccine Construct

To comprehensively map the antigenic landscape of the vaccine construct, a dual approach was employed for B‐cell epitope prediction. Initially, sequence‐based prediction of linear B‐cell epitopes was conducted using the BepiPred‐3.0 server (https://services.healthtech.dtu.dk/services/BepiPred‐3.0/) [39]. This advanced algorithm utilizes a protein language model to predict epitope propensity directly from the primary amino acid sequence. A predefined threshold of 0.15 was applied to identify residues with a high probability of acting as B‐cell epitopes. These predicted linear segments were subsequently mapped onto the 3D structure to visualize their spatial clustering and confirm their surface accessibility.

Furthermore, to identify potential discontinuous B‐cell epitopes within the folded chimeric protein, the refined tertiary structure of the vaccine construct was subjected to analysis using the DiscoTope‐3.0 web server (https://services.healthtech.dtu.dk/service.php?DiscoTope‐3.0). Unlike previous versions dependent solely on geometric features, this tool integrates inverse folding structural representations extracted from the ESM‐IF1 protein language model with a positive‐unlabeled (PU) learning ensemble strategy. This methodology is particularly robust for identifying epitopes on computational models, maintaining state‐of‐the‐art performance on AlphaFold‐predicted and relaxed structures compared to experimentally solved antigens. The 3D PDB file of the vaccine was used as input, and the prediction derived propensity scores for each residue based on spatial neighborhood and surface accessibility [40]. To mitigate potential biases related to antigen size, calibrated propensity scores were utilized, which normalize predictions based on antigen length, and surface area. A moderate confidence threshold of 0.90 (default) was selected to define positive epitope residues, corresponding to an expected recall of approximately 50% [40].

### 2.11 Tertiary Structure Refinement and Validation

AlphaFold 3 was utilized to predict the 3D structures of designed protein sequences (https://alphafold.ebi.ac.uk/). This server uses highly advanced deep learning algorithms, incorporating an attention‐based neural network architecture (Evoformer) to predict biomolecular structures and interactions with exceptional accuracy [41]. This server represents a significant breakthrough in the prediction of biomolecular structures. AlphaFold 3 has significantly improved prediction accuracy compared to its predecessors, particularly in predicting protein–ligand and protein–nucleic acid interactions, often outperforming specialized tools in these areas [42].

The refinement of 3D structures was enabled using the GalaxyRefine server (http://galaxy.seoklab.org/) [43]. This platform utilizes a refinement approach that initiates with repeated side‐chain repack perturbations followed by short molecular dynamics (MD) relaxations, facilitating larger molecular motions. It has been instrumental in functional protein modeling studies, enhancing the quality of model structures previously generated through various prediction techniques. A notable demonstration of its efficacy was observed in the CASP (Critical Assessment of techniques for protein Structure Prediction) refinement targets, where GalaxyRefine blind predictions proved successful, showcasing its reliability and accuracy in protein structure refinement tasks [43].

The validation of the 3D vaccine model was conducted using ProSA‐web, ERRAT, and Verify3D. ProSA‐web (https://prosa.services.came.sbg.ac.at/prosa.php), a widely utilized web server, evaluates the quality of the 3D model. It assigns a score, indicating potential errors if the score deviates from the range typical for native proteins. ERRAT (http://services.mbi.ucla.edu/ERRAT/) assesses non‐bonded interactions within the structure, while Verify3D (https://servicesn.mbi.ucla.edu/Verify3D/) examines the atomic model's compatibility with its amino acid sequence, classifying the structure based on environmental context and comparison with known structures. Afterward, the refined 3D model was validated via Ramachandran plot analysis, different quality factors, and error plot analysis via the PDBsum server (https://www.ebi.ac.uk/thornton‐srv/databases/pdbsum/) [44].

### 2.12 Docking, MD, and Binding Profile of Designed Vaccine With the Immune Cell Receptor TLR2/TLR4

To identify an optimized configuration between the vaccine protein (ligand) and TLR receptors (TLR2 and TLR4), molecular docking methods were employed. Utilizing PROPKA 3.1 software (https://github.com/jensengroup/propka/), the molecular structures of both the ligand and the receptor TLR2 (PDB ID: 6NIG)/TLR4 (PDB ID: 4G8A) were initially adjusted by modifying charges through protonation or deprotonation and correcting the bonds. Given that *T. cruzi* circulates in the bloodstream, a physiological pH range of 7.2–7.4 was selected for accuracy. Subsequent atomic optimization of hydrogen geometry utilized CHARMM, a force field from MD simulations tailored for organic molecules, to enhance the precision of these calculations.

The ClusPro 2.0 server (https://cluspro.bu.edu/login.php) [45] facilitated this process, analyzing the interaction between the vaccine protein and TLR2 (PDB ID: 6NIG)/TLR4 (PDB ID: 4G8A). ClusPro utilizes an FFT correlation algorithm for rigid‐body docking, evaluating billions of putative conformations, followed by RMSD‐based clustering of the lowest‐energy models and energy minimization. It generates outputs for the top ten models, with the one exhibiting the lowest binding energy deemed the most favorable among the docked solutions. Finally, the residues involved in the interactions between the vaccine and target receptors were visualized and the figures were created using Discovery Studio [46], while the mapping of these interactions was performed using PDBsum [44].

MD simulation is an important technique for the optimization of receptor–ligand complexes. In this study, MD simulations were performed using the GROMACS software with the CHARMM force field to investigate the binding stability of the final complex. The simulation system was solvated in a dodecahedral box using the TIP3P water model [47], maintaining a minimum distance of 15 Å between the protein and the box boundary. The dodecahedral geometry was chosen for its efficiency, minimizing the number of water molecules required and reducing computational costs without compromising accuracy. To mimic physiological conditions, Na^+^ and Cl^−^ ions were added, achieving a concentration of 0.15 M. The equations of motion were integrated using the Leap‐Frog algorithm, balancing computational efficiency and accuracy [48]. Long‐range electrostatic interactions were calculated using the Particle‐Mesh Ewald (PME) method with a 1.2 nm cutoff and van der Waals interactions were also included [49]. System minimization was performed via the steepest descent algorithm over 10,000 steps, followed by two equilibration phases: Around 1 ns in the NVT ensemble to stabilize temperature and 1 ns in the NPT ensemble to equilibrate pressure. The production phase lasted 300 ns, and a 2.0 fs integration time step was employed for the simulation of the TLR2/TLR4–vaccine complex. Temperature was maintained at 310.15 K using the V‐rescale thermostat [50], and pressure was controlled with the Parrinello–Rahman barostat [51]. Key simulation parameters monitored during this process included the root mean square deviation (RMSD) and the root mean square fluctuation (RMSF), which are fundamental for assessing the stability and dynamics of the system throughout the simulation.

### 2.13 Normal Mode Analysis

To evaluate the stability and dynamic behavior of the docked vaccine–TLR2 and TLR4 complexes, normal mode analysis (NMA) was performed using the iMOD server (iMODS) (http://imods.chaconlab.org) [52]. This platform uses NMA in internal (dihedral) coordinates to accurately model the collective functional motions inherent to biological macromolecules. In addition, it facilitates the generation of plausible transition pathways between two homologous structures, so that large macromolecules can also be considered [52].

### 2.14 Codon Optimization and In Silico Cloning

To express the chimeric protein in an expression system, the amino acid sequence was reverse translated using the backtranseq program from the EMBOSS 6.0.1 software suite (https://www.ebi.ac.uk/Tools/st/emboss_backtranseq/). The EMBOSS software analysis package efficiently handles data in various formats and facilitates the seamless retrieval of sequence data from the web [53]. To generate the vaccine cDNA sequence required for effective expression in the K‐12 strain, the Java Codon Adaptation Tool (JCat), which can be accessed at https://www.jcat.de/, was used for codon optimization and back translation. It was also evaluated with the Codon Adaptation Tool to predict the appropriate expression in vector translation and cloning efficiency. In addition, the SnapGene tool was used to introduce the optimized multiepitope vaccine sequence into the pET‐28a (+) vector [54].

### 2.15 Immune Stimulation

The C‐ImmSim server (https://kraken.iac.rm.cnr.it/C‐IMMSIM/) was used to visualize the immunogenic profiles and immune response of the vaccine prototype. The online server C‐ImmSim uses agent‐based modeling to describe the immunogenicity and immune response profile of chimeric peptides [55]. PSSM for peptide prediction, derived from machine learning techniques for the prediction of immune interactions, serve as the basis for the model. Two injections of the targeted preventive vaccine profile were administered 6 weeks apart. We ran our simulation for 1000 time steps or over 12 months (one time step corresponds to about 8 h) [56].

## 3 Results and Discussion

Our research on vaccine candidates for Chagas disease has focused on two key proteins, concentrating on their different roles during the various stages of the parasite's life cycle. These antigens include the ASP‐2, which is encoded by a gene family that is mainly expressed in the amastigote form, and gp82, which is encoded by a gene family that is mainly expressed in the trypomastigote form [57].

Our strategic approach aims to develop a vaccine targeting these surface glycoproteins, which are crucial not only for their different roles in parasite‐host interactions, but also for their ability to elicit strong immune responses. These responses involve T cells and B cells, key components in combating the different morphological stages of the parasite. We have identified epitopes of gp82/ASP‐2 based on human T‐cell and B‐cell epitopes, characterized by their high antigenicity, immunogenicity, non‐allergenicity, non‐toxicity, and broad population coverage (Figure 1). These epitopes have been shown to induce robust protective immunity in combination with adjuvants and linkers.

The current body of research shows that multi‐epitope vaccines targeting both amastigote and trypomastigote forms can elicit robust immune responses, especially when formulated with potent adjuvants, and provide protection against *T. cruzi* infection in animal models such as dogs and mice [58]. These findings are significant because they address the chronic phase, where current drugs like benznidazole have limited efficacy due to toxicity or incomplete parasite clearance.

Studies show that gp82 plays a crucial role in host cell invasion by inducing signaling cascades that lead to actin cytoskeleton disruption and lysosome exocytosis, facilitating parasite internalization [59, 60]. Furthermore, opsonization of *T. cruzi* metacyclic trypomastigotes with gp82‐specific antibodies reduces parasite infectivity, highlighting the potential of gp82 as a vaccine target for Chagas disease. These findings collectively suggest that epitopes gp82 could be a promising approach in combating *T. cruzi* infections [61]. Despite these advances, translation to real‐world human populations faces challenges due to parasite diversity, diagnostic limitations, and the need for region‐specific validation.

Studies have shown that immunization with chimeric proteins containing immunogenic regions from ASP‐2 can induce robust protection against *T. cruzi* in mice and dogs [10, 62]. Recent clinical research has focused on evaluating the efficacy of such vaccines in natural hosts (notably dogs) and exploring their potential as adjuncts to chemotherapy. A randomized field trial in dogs demonstrated that a recombinant protein vaccine (Tc24‐C4 and TSA1‐C4 with MPLA adjuvant) significantly reduced parasite burden and prevented cardiac alterations, supporting further development for both veterinary and human use. Immunological analysis revealed reprogramming of T cell responses (Th1/Th2/Th17 balance) as a mechanism of action [58]. Diagnostic advances and serological monitoring are also critical for evaluating vaccine efficacy and treatment outcomes

### 3.1 Acquisition of Protein Sequences

The complete genome sequences of proteins ASP‐2 and gp82 were obtained from NCBI. The MAFFT alignment tool was used for sequence alignment of ASP‐2 and gp82 sequences to distinguish highly conserved regions. Finally, for ASP‐2, the consensus sequence was derived from the 13 identified sequences using the JALVIEW 2.11.1.4 program. Similarly, the consensus sequence for gp82 was obtained from the 36 identified sequences using JALVIEW 2.11.1.4 and the sequences are fully conserved in the selected isolates of proteins of different evolutionary forms. This suggests that selected epitopes, when used for the development of multivalent epitope vaccines, could provide broad therapeutic options for different evolutionary forms of *T. cruzi*.

### 3.2 CTL Epitope Selection

For the ASP‐2, NetMHCpan4.1 successfully identified 143 9‐mer epitopes across the 35 evaluated HLA alleles. Similarly, for the antigen gp82, NetMHCpan identified 116 9‐mer epitopes across 35 HLA alleles, which demonstrates the potential for these epitopes to elicit a broad immune response in individuals with diverse genetic backgrounds [63].

The proteasome degrades vaccine or pathogen peptides in the cytoplasm by unfolding the protein and cleaving it down into smaller fragments (peptides) [63]. The protein transporter (TAP) transports them into the endoplasmic reticulum. The MHC I groove is only stable for peptides that are the right size [64]. These properties are assessed by the NetCTL 1.2 server, which also analyzes MHC class I binding, TAP transport efficiency and proteasomal cleavage. NetCTL1.2 detected 169 epitopes for protein gp82 and 182 epitopes for ASP‐2 in the 12 HLA supertypes.

We found 81 peptides for ASP‐2 and 89 for gp82 that were shared by both programs (see Tables S1 and S2). The most promising CTLs were chosen based on their antigenicity, immunogenicity, allergenicity, and toxicity. Following these criteria, we screened 15 gp82 and 11 ASP‐2 (Table 1).

**TABLE 1:** Selected CTL epitopes for the final vaccine in ASP‐2 and gp82 provided by NetMHCIpan and NetCTL servers.

Pos.	CTL Epitope	Alleles	Supertypes	Antigenicity	Allergenicity	Immunogenicity	Toxicity	Pro‐inflammatory inducing
365	MSQPGVRLY (ASP‐2)	9	3	0.6795	Non‐allergen	0.06030	Non‐toxin	Positive
531	TPTAGLVGF (ASP‐2)	8	1	0.5301	Non‐allergen	0.12917	Non‐toxin	Positive
445	ETLANALLY (ASP‐2)	3	3	0.5405	Non‐allergen	0.05046	Non‐toxin	Positive
157	VAEVNATTW (ASP‐2)	2	1	0.8398	Non‐allergen	0.16001	Non‐toxin	Positive
603	QYSFVNHRF (ASP‐2)	2	1	0.6681	Non‐allergen	0.15575	Non‐toxin	Positive
117	MVAFAEGLF (ASP‐2)	1	1	0.9191	Non‐allergen	0.28497	Non‐toxin	Positive
317	CLDPRITEW (ASP‐2)	1	1	0.9106	Non‐allergen	0.26298	Non‐toxin	Positive
163	TTWRAHTVI (ASP‐2)	1	1	1.1920	Non‐allergen	0.24941	Non‐toxin	Positive
166	RAHTVIGSR (ASP‐2)	1	1	1.3874	Non‐allergen	0.14698	Non‐toxin	Positive
596	NSREDNRQY (ASP‐2)	1	3	1.7903	Non‐allergen	0.10906	Non‐toxin	Positive
444	NETLANALL (ASP‐2)	1	1	0.6534	Non‐allergen	0.05999	Non‐toxin	Positive
143	TSSAVVAEY (GP‐82)	9	5	0.6132	Non‐allergen	0.15625	Non‐toxin	Positive
380	VEALITATI (GP‐82)	4	1	0.9951	Non‐allergen	0.22338	Non‐toxin	Positive
41	RRVFDSTIL (GP‐82)	4	2	0.5852	Non‐allergen	0.10759	Non‐toxin	Positive
654	TPEVRGAEI (GP‐82)	3	1	1.3338	Non‐allergen	0.24786	Non‐toxin	Positive
275	VLMEDGTLV (GP‐82)	3	1	1.3338	Non‐allergen	0.12353	Non‐toxin	Positive
281	TLVFPVIAF (GP‐82)	2	1	1.2479	Non‐allergen	0.29444	Non‐toxin	Positive
556	FVNYNFTLV (GP‐82)	2	1	0.7731	Non‐allergen	0.12436	Non‐toxin	Positive
555	TFVNYNFTL (GP‐82)	2	2	1.0138	Non‐allergen	0.11868	Non‐toxin	Positive
244	ITWGTPTSL (GP‐82)	2	2	0.9477	Non‐allergen	0.06946	Non‐toxin	Positive
507	SGDAWIDDY (GP‐82)	1	1	1.2497	Non‐allergen	0.41923	Non‐toxin	Positive
563	LVATVIVHK (GP‐82)	1	1	0.7561	Non‐allergen	0.27098	Non‐toxin	Positive
276	LMEDGTLVF (GP‐82)	1	3	0.4810	Non‐allergen	0.13912	Non‐toxin	Positive
8	VMAPRTHNR (GP‐82)	1	1	0.6569	Non‐allergen	0.11200	Non‐toxin	Positive
48	ILLLLVTTM (GP‐82)	1	3	0.8106	Non‐allergen	0.06874	Non‐toxin	Positive
3	SRVAAVMAP (GP‐82)	1	1	0.6827	Non‐allergen	0.00439	Non‐toxin	Positive

In our research, we discovered the 9‐amino acid CTL epitope ASP‐2^531‐539^, which closely matched the reported 10 amino acid epitope by Castro et al. [10]. Further investigation revealed that this epitope is promiscuous with excellent antigenicity, high immunogenicity, non‐toxicity, and no allergenic properties. However, despite these promising features, some CTL epitopes like RQYSFVNHR and TEWETGQIL were excluded due to their low antigenicity scores of 0.3188 and 0.09, respectively, falling below our acceptance threshold of 0.4.

### 3.3 HTL Epitope Selection

NetMHCIIpan discovered 92 15‐unit epitopes for the 11 HLAs analyzed by ASP‐2, whereas NetMHCII identified a total of 135 such epitopes for all provided HLAs. In the case of gp82, NetMHCII found 155 instances of these epitopes across all HLAs in the program, while NetMHCIIpan identified only 79 for the evaluated set of 11 HLAs. The comparative analysis between NetMHCII and NetMHCIIpan revealed a significant overlap in epitope predictions for each protein individually, with 36 common peptides identified for ASP‐2 and 40 for gp82, supporting the effectiveness of both tools in identifying epitopes within these antigens (Tables S3 and S4). Subsequent analysis of HTL epitopes identified three with strong antigenicity, high immunogenicity, and predicted safety properties, as they were classified as non‐toxic, non‐allergenic, and pro‐inflammatory (Table 2), supporting their potential as promising vaccine candidates.

**TABLE 2:** Selected HTL epitopes for the final vaccine in ASP‐2 and gp82 provided by NetMHCII and NetMCHIIpan servers.

Pos.	HTL Epitope	Alleles	Antigenicity	Allergenicity	Immunogenicity	Toxicity	Pro‐inflammatory inducing
735	TNVFLYNRPLSVGEL (ASP‐2)	5	0.7536	Non‐allergen	92.3299	Non‐toxin	Positive
736	NVFLYNRPLSVGELK (ASP‐2)	4	0.8399	Non‐allergen	93.4249	Non‐toxin	Positive
695	ADELRMVKKRKPEDK (GP‐82)	3	0.6406	Non‐allergen	55.5337	Non‐toxin	Positive

### 3.4 BL Epitope Selection

B cell epitopes bind to B cell precursors and trigger humoral immune responses [65]. Computational methods have been developed to predict both linear and discontinuous B cell epitopes, facilitating the development of vaccines and the advancement of antibodies [66].

Several B cell epitopes against conserved ASP‐2 and gp82 proteins were found by IEDB and BCEPred, two B cell epitope prediction servers. The predictions from both methods were concurrently aligned and 10 (gp82) and 8 (ASP‐2) epitopes were selected (see Tables S5 and S6). In addition, 4 (ASP‐2) and 5 (gp82) epitopes that exhibited high scores as well as high antigenicity, non‐allergenicity, and positive induction of pro‐inflammatory cytokines were selected as final epitopes (Table 3).

**TABLE 3:** Selected linear/continuous B‐cell (LBL) epitopes for the final vaccine in ASP‐2 and gp82 provided by IEDB and BCEPred servers.

Linear B cell epitopes	length	Antigenicity	Allergenicity	Toxicity	Pro‐inflammatory inducing
AGLVGFLSN (ASP‐2)	9	1.6103	Non‐allergen	Non‐toxin	Positive
GQILMIVQCG (ASP‐2)	10	0.9079	Non‐allergen	Non‐toxin	Positive
GTLVFPLMVS (ASP‐2)	10	0.7064	Non‐allergen	Non‐toxin	Positive
DVTVTNVFLYNRPLSVG (ASP‐2)	17	0.5098	Non‐allergen	Non‐toxin	Positive
AGLVGLLSN (GP‐82)	9	1.1596	Non‐allergen	Non‐toxin	Positive
GTLVFPVIAF (GP‐82)	10	1.0430	Non‐allergen	Non‐toxin	Positive
LLGAVLAEPISTLFIGLSY (GP‐82)	19	0.9440	Non‐allergen	Non‐toxin	Positive
VSRALLLLLMLLGLW (GP‐82)	15	0.9366	Non‐allergen	Non‐toxin	Positive
AFVSPSLVSAGGVLAAF (GP‐82)	17	0.5502	Non‐allergen	Non‐toxin	Positive

### 3.5 Population Coverage

The variability of HLA alleles across different regions, influenced by environmental factors, underscores the importance of achieving extensive population coverage in vaccine development [67, 68, 69]. As demonstrated in Table 4, the population coverage analysis revealed that the class combined (MHC I and II) epitopes selected in this study represent 61.98% of the Latin American population. The regions coverage rates, highlighting Central America (13.18%), Mexico (83.1%), and South America (89.66%). Achieving comprehensive population coverage is essential to ensure that a significant portion of the global population benefits from a single, effectively designed vaccine [69].

**TABLE 4:** Population coverage of the class combined coverage of epitopes included in the vaccine construct for Latin America.

Population/Area	Coverage^a^	Average_hit^b^	Pc90^c^
Central America	13.18%	0.22	0.12
Mexico	83.1%	3.16	0.59
South America	89.66%	3.59	0.97
Average	61.98	2.32	0.56
Standard deviation	34.61	1.5	0.35

^a^projected population coverage.

^b^average number of epitope hits/HLA combinations recognized by the population.

^c^minimum number of epitope hits/HLA combinations recognized by 90% of the population.

### 3.6 Vaccine Sequence Construction

A chimeric vaccine was meticulously developed with a strategic overlap of 38 epitopes, which included 9 B‐cell epitopes, 26 CTL epitopes, and 3 HTL epitopes. The adjuvants used were β‐defensin‐4 (NCBI ID: NP_001192195) and the 50S ribosomal protein L7/L12 (UniProt ID: P9WHE3) and were attached to the N‐terminal of the vaccine sequence through the EAAAK linker to induce a specific immune response. In addition, the linkers AAY and KK were used to connect overlapping CTL epitopes, HTL epitopes, and B‐cell epitopes, respectively. This structural arrangement mirrors multi‑epitope vaccine designs where EAAAK connects adjuvants to the epitope string, keeping domains separated and structurally stable. AAY, KK, and EAAAK linkers promoted proper separation of epitopes, prevented junctional epitopes, and supported efficient processing by proteasome, TAP, and lysosomal pathways, enabling effective MHC I/II presentation and preserving accessibility of B‑cell epitopes. This architecture mirrors successful designs reported for *Brucella* spp., *Goatpox* virus, *Klebsiella aerogenes*, *Treponema pallidum*, and mRNA fungal vaccines, which similarly employ AAY for CTL epitopes, KK for B‐cell epitopes, and adjuvant–EAAAK fusions to preserve structural integrity and improve immunogenicity [70, 71, 72, 73, 74]. The resultant vaccine, comprising 724 amino acids, effectively demonstrated the arrangement of diverse epitopes and linkers.

### 3.7 Physiochemical Properties, Antigenicity, Allergenicity, and Solubility Potential of the Designed Vaccine

The analysis from the Protparam server revealed that the MW of the vaccine structure was 78.40 kDa, with a theoretical isoelectric point (pI) of 9.56, indicating an alkaline and stable structure with an instability index of 27.38 (an index below 40 is considered stable) [75]. Furthermore, the aliphatic index of the vaccine sequence was 104.72, and the GRAVY value was 0.262, suggesting that the vaccine construct is thermostable and hydrophobic. The structure exhibited a half‐life of 30 h in mammals, estimated to exceed 20 h in yeast and over 10 h in *E. coli*. The solubility of the vaccine construct was confirmed by a solubility score of 0.840063 from the SOLpro server. The antigenicity and allergenicity assessments of the final sequence of the candidate vaccine structure indicated its antigenic nature (0.5795 ‐ Vaxijen score) and non‐allergenic behavior. These findings demonstrate that the preparation possesses basic, stable, thermostable, and hydrophobic characteristics, which, apart from hydrophobicity, are favorable attributes for a vaccine (Table 5).

**TABLE 5:** Basic characteristics of the vaccine structure designed against *Trypanosoma cruzi*.

Property	Result
Allergenicity (AllerTOP v2.0)	Non‐allergic
Antigenicity (VaxiJen v2.0), score	Antigen, 0.5795
Predicted solubility upon overexpression (scratch), score	Soluble, 0.840063
Aliphatic index	104.72
Stability index (II)	27.38
Expression vector	*Escherichia coli*
Theoretical pI	9.56
Number of Amino acids	724
Half‐life	30 h (mammalian reticulocytes, in vitro) >20 h (yeast, in vivo) >10 h (*E. coli*, in vivo)
Formula	C_3590_H_5790_N_920_O_985_S_25_
GRAVY	0.262

### 3.8 Secondary and Tertiary Structure Prediction of the Vaccine Construct

The Prabi servers facilitated the prediction of the secondary structure of the vaccine construct using the PSIPRED and SOPMA tools. The PSIPRED server predicted 18.1% alpha helices, 30.66% extended strands, and 51.24% random coils (Figure 2A). Conversely, SOPMA predicted that the secondary structure of the vaccine would consist of 43.65% alpha helix, 22.65% extended strands, and 23.34% random coils (Figure 2B). Furthermore, the Prabi server predicted 50.28% alpha helices, 13.95% extended strands, and 35.77% random coils (Figure 2C). A high percentage of random coils indicated the greater flexibility of the vaccine structure.

**FIGURE 2:**
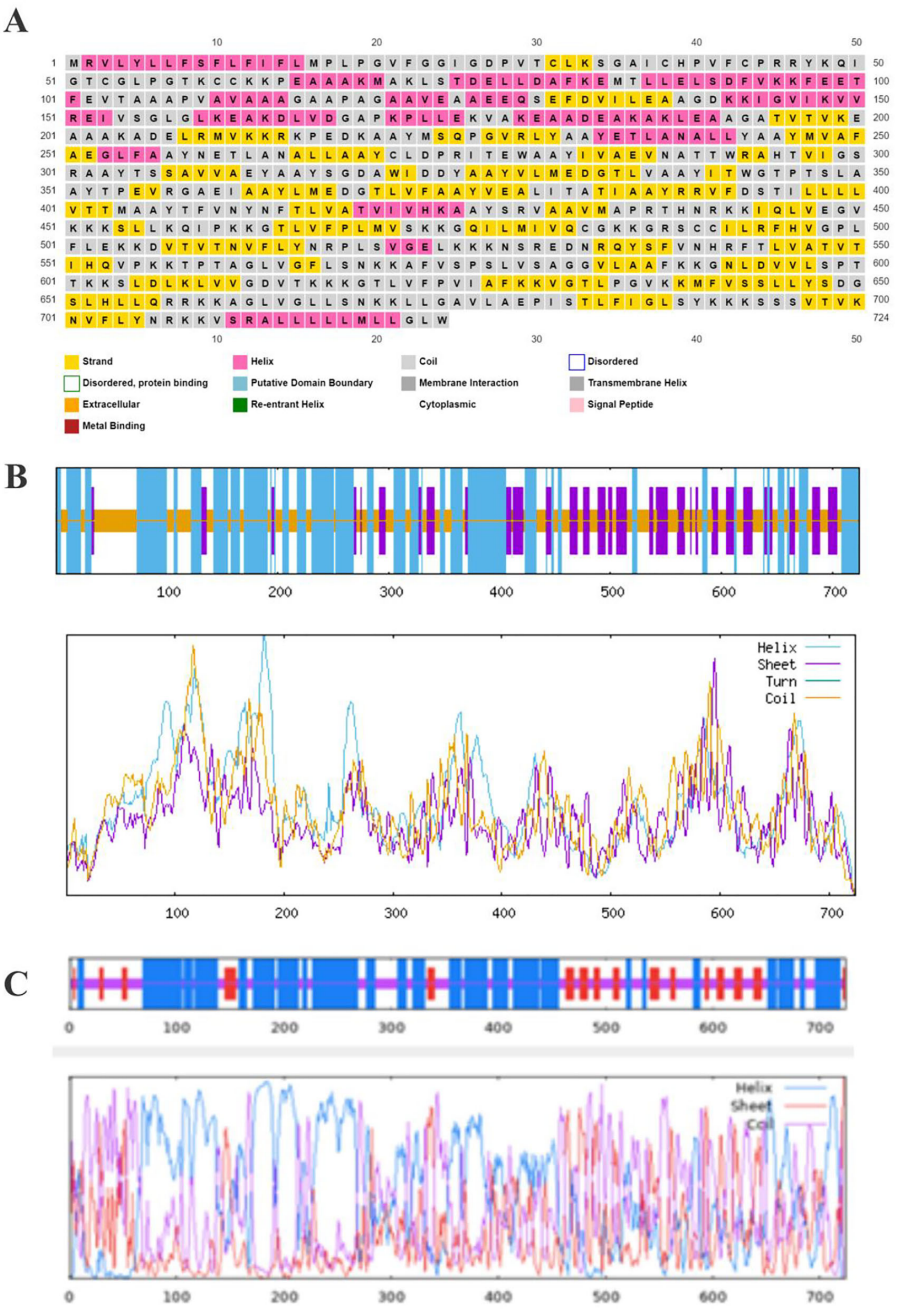
Predicted schematic image of the secondary structure of the designed vaccine by: (A) PSIPRED server, (B) SOPMA server, (C) Prabi server.

The observed discrepancies in the percentages of secondary structure elements are expected, as these tools utilize distinct algorithms: PSIPRED employs ANNs based on PSI‐BLAST profiles, SOPMA relies on a consensus prediction method, and Prabi typically uses the GOR IV information theory‐based approach [37, 38, 76]. While these sequence‐based (1D) predictions provide preliminary estimates, they do not fully account for tertiary folding energy.

### 3.9 Refinement and Validation of Final Vaccine Construct

The final vaccine construct underwent refinement to correct errors predicted by the initial model. The top model was selected and optimized by GalaxyRefine. The estimated RMSD for the selected model was 0.53. Galaxy web server was used to refine model 1 obtained from Alphafold3 server (Figure 3).

**FIGURE 3:**
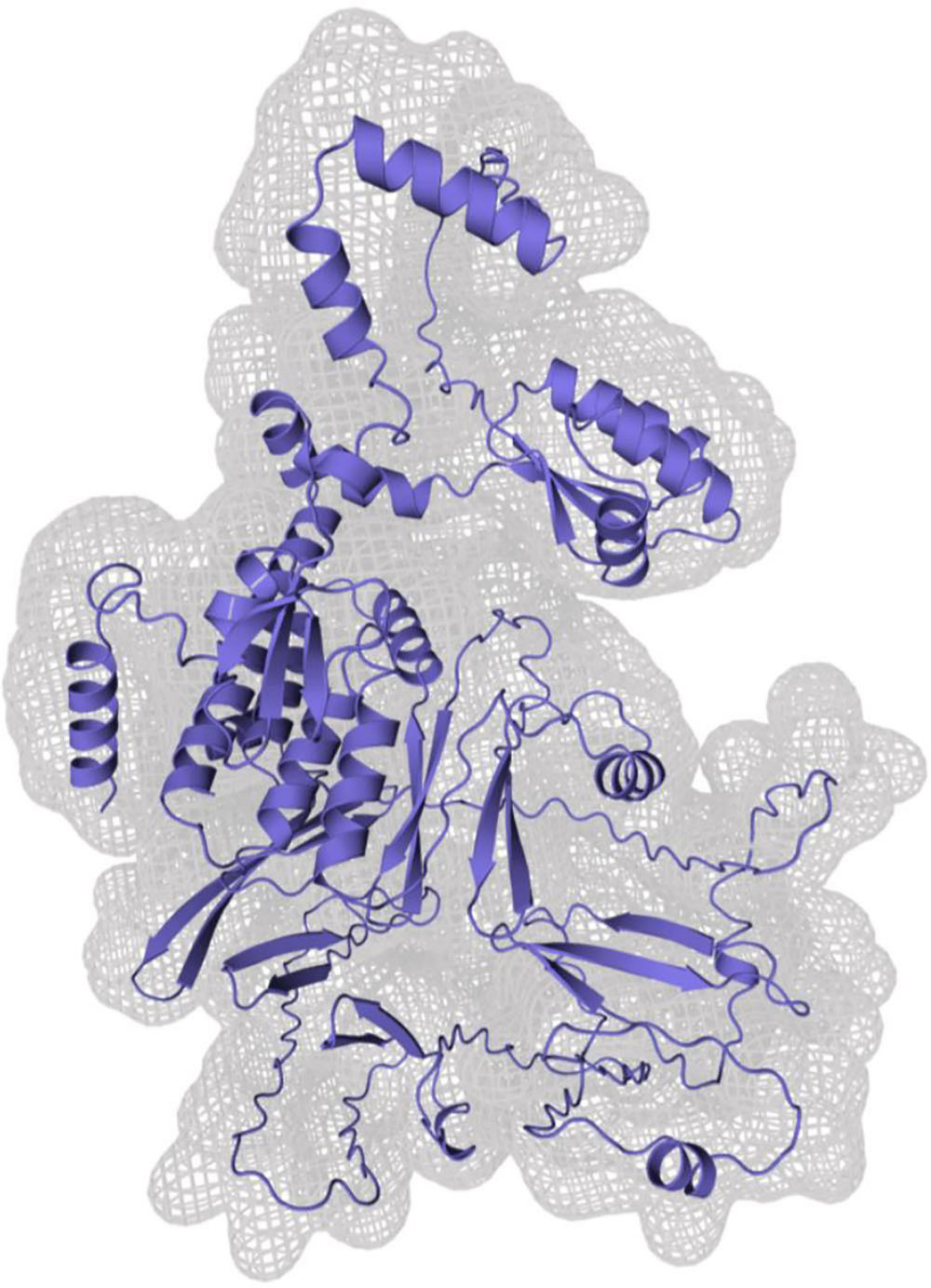
Refined three‐dimensional (3D) structural model of the newly designed multi‐epitope vaccine construct.

The refinement of the structure improved protein structure as evidenced by a Ramachandran plot showing an increase from 89.9% to 98.5% of residues in the favored region (Figure 4). Subsequent validation of the refined model, a crucial step following homology modeling, involved further Ramachandran plot analysis (Figure 4). Results indicated 26.09% of residues in the allowed region, with outliers constituting 0.34%. Moreover, the ERRAT score was 96.70, significantly higher than the threshold of 80 (Figure S1A). The overall integrity and energy deviation of the vaccine structure were assessed using a *Z*‐score. A positive ProSA value typically suggests a suboptimal structure, while *Z*‐scores above −4 denote an average structure. Our vaccine achieved a *Z*‐score of −2.41 before refinement and −3.28 after refinement, indicating it falls within the desirable range of structural integrity, as illustrated in Figure S2.

**FIGURE 4:**
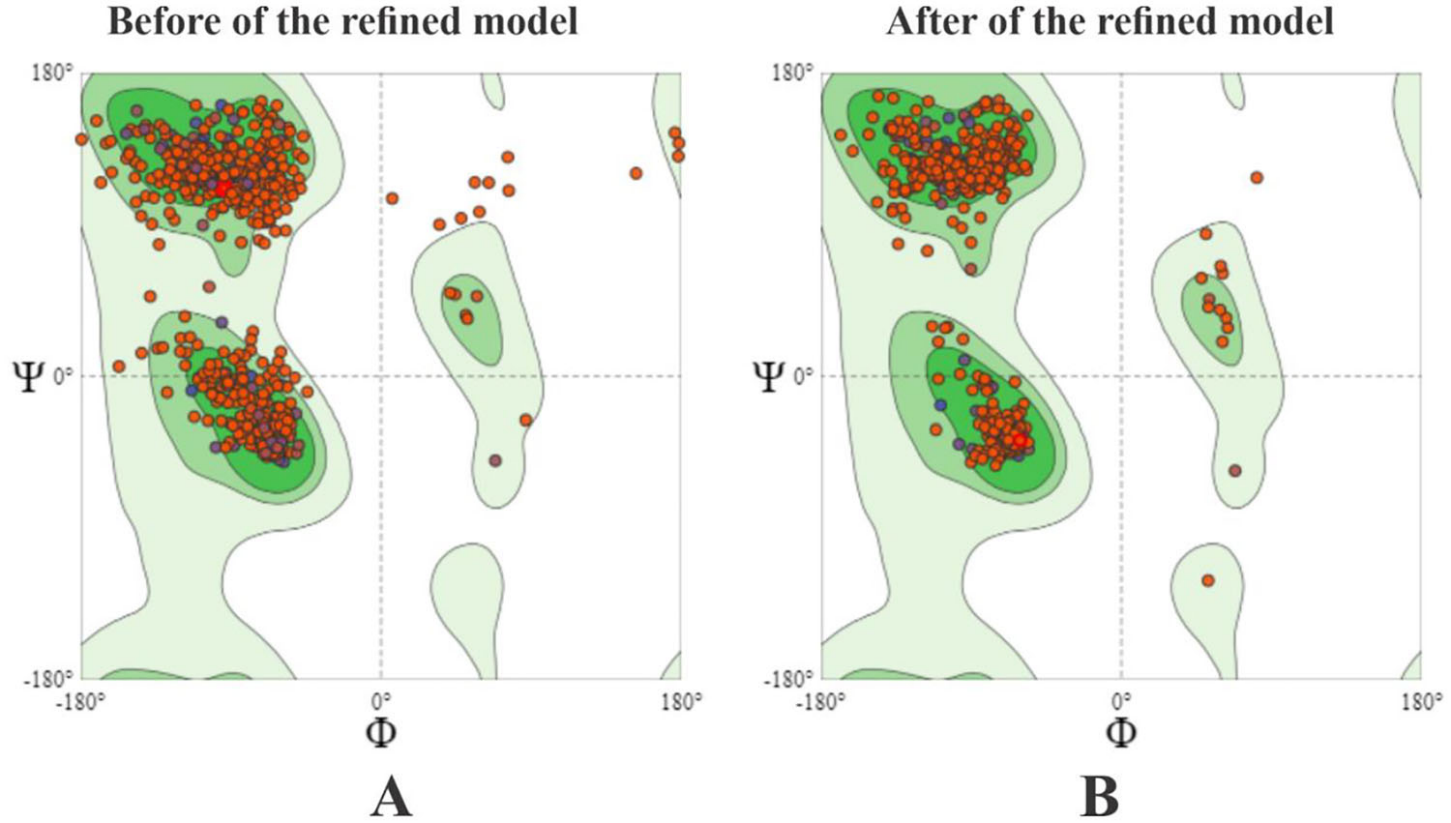
Ramachandran plot analysis of the vaccine construct (A) before and (B) after structural refinement.

### 3.10 Prediction and Structural Mapping of Discontinuous B‐Cell Epitopes

The induction of a robust humoral immune response relies on the efficient recognition of B‐cell epitopes by circulating antibodies and B‐cell receptors [77]. To identify these critical antigenic regions within the designed construct, we evaluated the sequence using the BepiPred‐3.0 prediction algorithm. As illustrated in the epitope propensity plot (Figure 5), numerous segments across the protein sequence scored above the stringent predefined threshold of 0.15. Notably, highly antigenic hotspots, indicated by prominent yellow and orange peaks reaching scores up to ∼0.26, were identified distributed throughout the sequence, with prominent clusters observed around positions 200, 400–500, and 600–700 [78].

**FIGURE 5:**
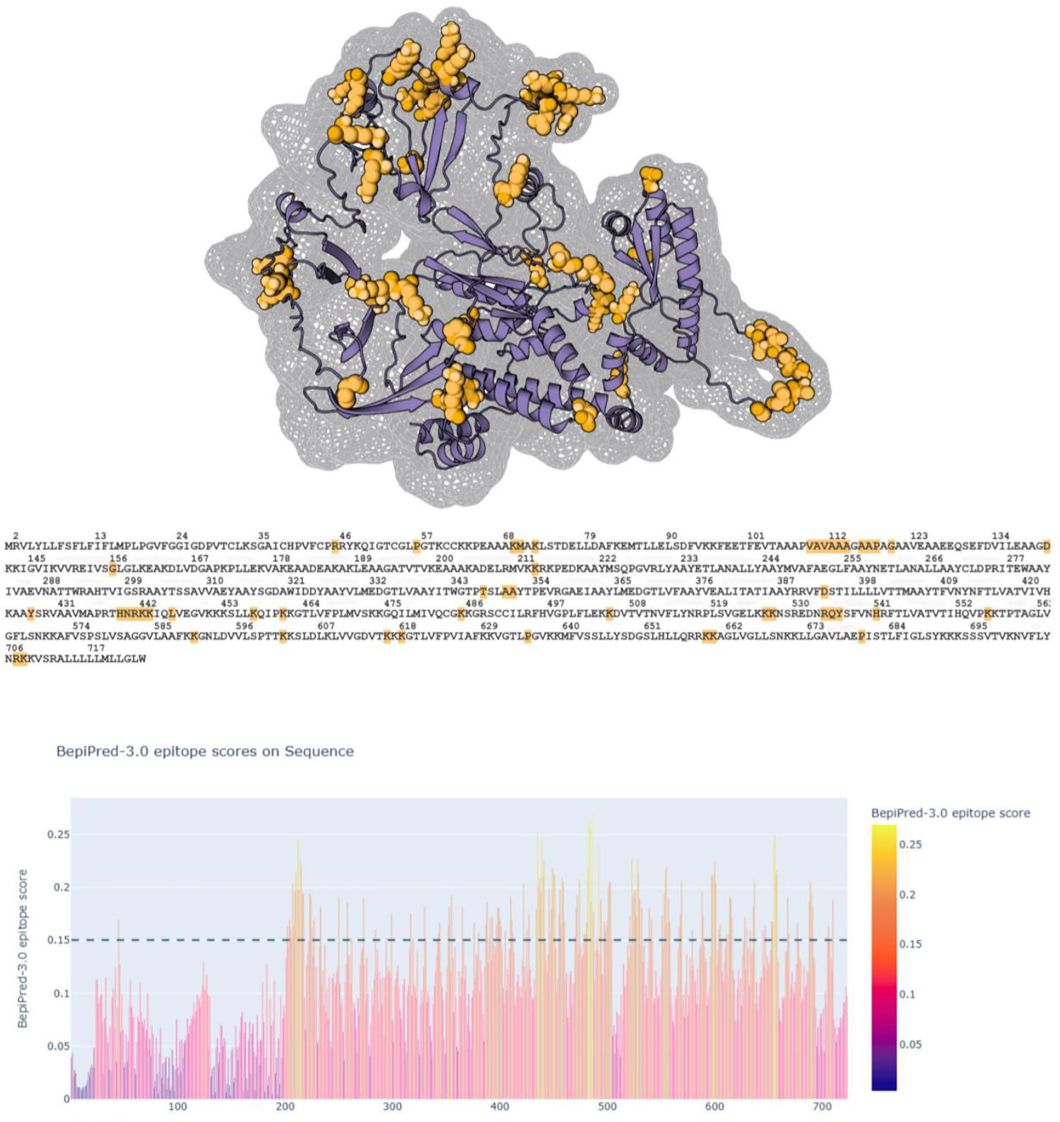
Prediction and mapping of B‐cell epitopes on the designed vaccine construct. (Top) 3D structural model of the protein with predicted discontinuous B‐cell epitopes highlighted as orange spheres, demonstrating high surface accessibility. (Middle) Primary amino acid sequence mapping the exact locations of the antigenic residues (highlighted in orange). (Bottom) BepiPred‐3.0 epitope propensity score plot across the sequence length, with the dashed line indicating the threshold of 0.15. Peaks in yellow/orange represent regions with the highest probability of acting as B‐cell epitopes.

To confirm the structural viability and accessibility of these predicted regions, the identified residues were mapped directly onto both the primary sequence (Figure 5) and the tertiary structure of the protein (Figure 5). In the 3D model, the residues comprising the predicted discontinuous B‐cell epitopes are rendered as orange spheres. The spatial visualization clearly demonstrates that these antigenic residues are predominantly clustered on the outer exposed surface of the folded protein. This high degree of surface accessibility is a critical attribute for conformational (discontinuous) epitopes, as it ensures they are not buried within the hydrophobic core, remaining sterically available for direct interaction with paratopes of antibodies. Consequently, the prominent surface display of these epitopes strongly supports the construct's structural design and its potential to effectively stimulate humoral immunity [78].

### 3.11 Docking, MD, Normal Modes, and Binding Profile of Final Vaccine Construct in Immune Cell Receptors (TLR2/TLR4)

Investigations into our vaccine prototype interactions with specific immune receptor molecules are critical for advancing our understanding of immunological defense mechanisms. Our findings indicate that the dynamics of vaccine–receptor interactions play a significant role in enhancing the vaccine's ability to modulate immune responses, highlighting their importance in vaccine effectiveness and design optimization.

In our research, we employed molecular docking techniques on two specific toll‐like receptors (TLRs), highlighting their significance in the synthesis of agonists. These TLRs demonstrate a specialized ability to detect a range of molecular signatures presented by invasive pathogens, thereby orchestrating the primary signaling pathways required for a robust immune response to trypanosomiasis [79, 80]. The focus of our vaccine development was on selecting both membrane‐bound and secreted proteins from *T. cruzi*, which have shown potent efficacy in activating TLR2 across species. This is noteworthy for its role in eliciting TLR4 mediated pro‐inflammatory responses against the disease [81].

Utilizing ClusPro for our docking analysis, we generated and evaluated 30 predictive models for each complex vaccine‐TLR. Among these models, only one model was chosen on the basis of different energy parameters for further study (Figure 6). Out of the top 30 predicted models for TLR2, Model Number 2 was selected due to its lowest energy score of −1360.4. Similarly, for TLR4, Model Number 19 stood out from the 30 candidates with the lowest energy score of −1278.7.

**FIGURE 6:**
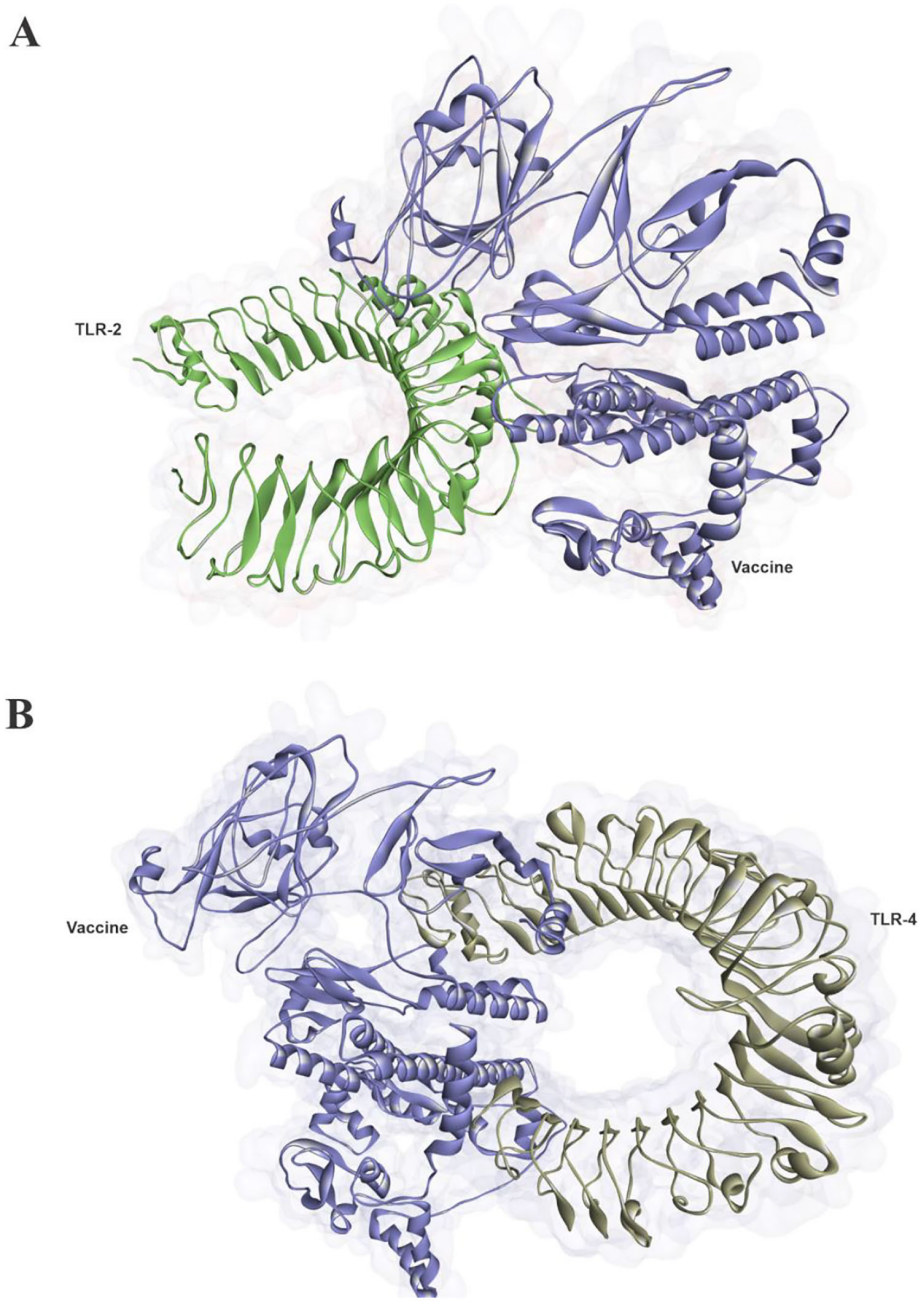
Molecular docking of the vaccine with (A) TLR2 and (B) TLR4.

It is well recognized that the accuracy of scoring functions is often insufficient, necessitating the use of more reliable methods to identify the optimal pose among docking results [82]. To obtain more realistic insights in our vaccine–protein docking study, it was essential to consider the flexibility of the protein structure and account for all thermodynamic processes (such as enthalpy, entropy, and free energy changes) involved in the interaction of a molecule with a receptor in an aqueous environment through MD simulations [83, 84].

MD simulations were carried out for 300 ns to investigate the structural dynamics and stability of the TLR2–vaccine and TLR4–vaccine complexes. Both the RMSD and RMSF analyses were performed, using the alpha‐carbon atoms (Cα) of each structure as the basis for calculation. This approach provides a reliable measure of backbone stability and flexibility for both the receptors and the vaccine.

The RMSD graph, Figure 7A,B, derived from 300 ns of simulation, elucidates the stability of the TLR2–vaccine and TLR4–vaccine complexes. In all systems, the RMSD initially displayed significant swings, especially during the first 50 ns, after which the values steadied, signifying the attainment of dynamic equilibrium. The stabilization of the RMSD values following this interval indicates that both the TLR2 and TLR4 receptors, together with the vaccine, attained stable conformations. The RMSD values for the vaccine consistently exceeded those of the receptors in both systems, aligning with the hypothesis that the vaccine, as a more flexible molecular structure, would demonstrate more structural deviations. The stability noted throughout the simulation validates that the sampled conformations accurately represent an equilibrium state, providing a robust foundation for further investigation.

**FIGURE 7:**
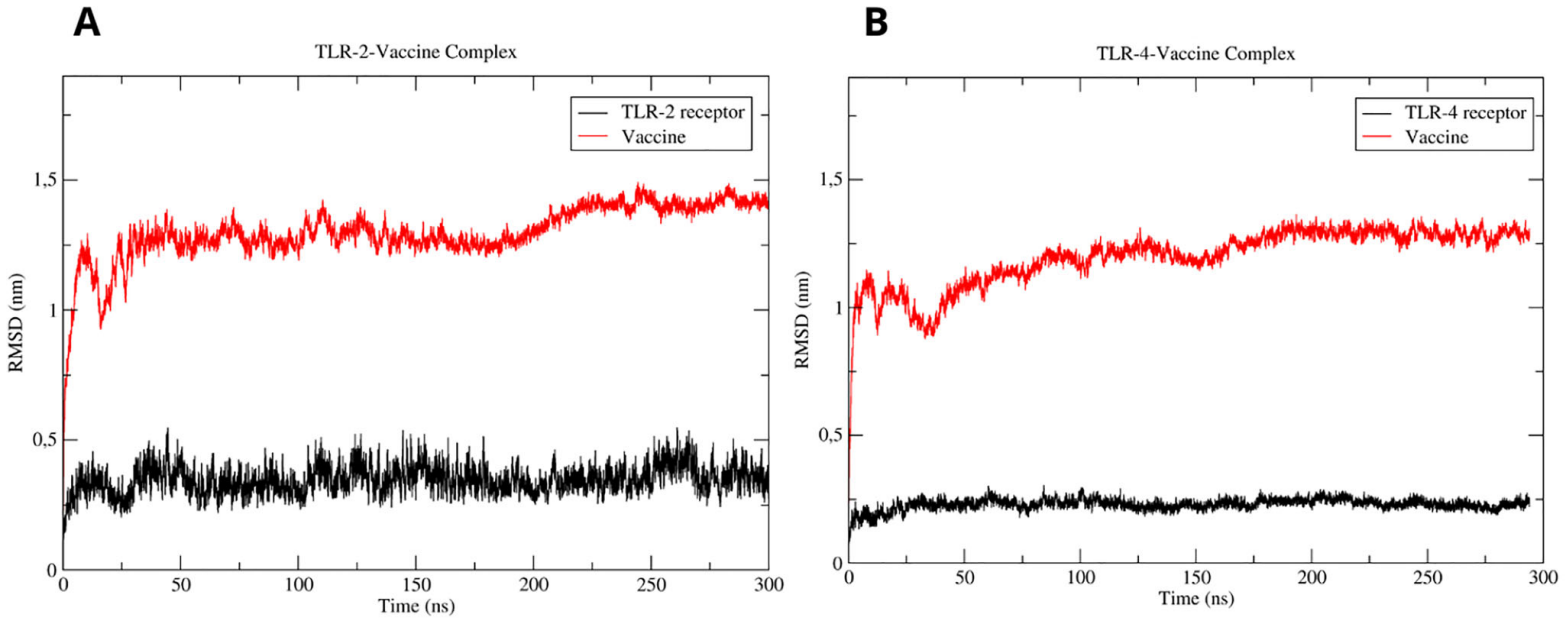
Root mean square deviation (RMSD) of TLR2‐Vaccine (A) and TLR4‐Vaccine (B) complexes in 300 ns molecular dynamics simulations. For both, the receptors are represented in black, and the vaccine is represented in red.

The RMSF analysis depicted in Figure 8A–D elucidates the flexibility of specific residues for both the TLR2 and TLR4 receptors, together with the vaccine in their respective complexes. Figure 8A,B present the RMSF values for the TLR2 and TLR4 receptors. Both receptors demonstrated minimal variations, with RMSF values remaining below 0.4 nm for most residues. Peaks in the RMSF graphs indicate flexible regions, such as loop regions and termini, which exhibit increased mobility. In TLR2 (Figure 8A), notable peaks are observed around residues 240–260 (Region ii), 290–310 (Region iii), and at the C‐terminus (Region iv), indicating enhanced flexibility in these areas. Similarly, for TLR4 (Figure 8B), increased flexibility is observed around residues 150–170 (Region i), 230–250 (Region ii), and 330–350 (Region iii). These flexible regions correspond closely to the areas highlighted in red in the b‐factor surface cartoons, with the most intense red coloration indicating the highest levels of atomic fluctuation.

**FIGURE 8:**
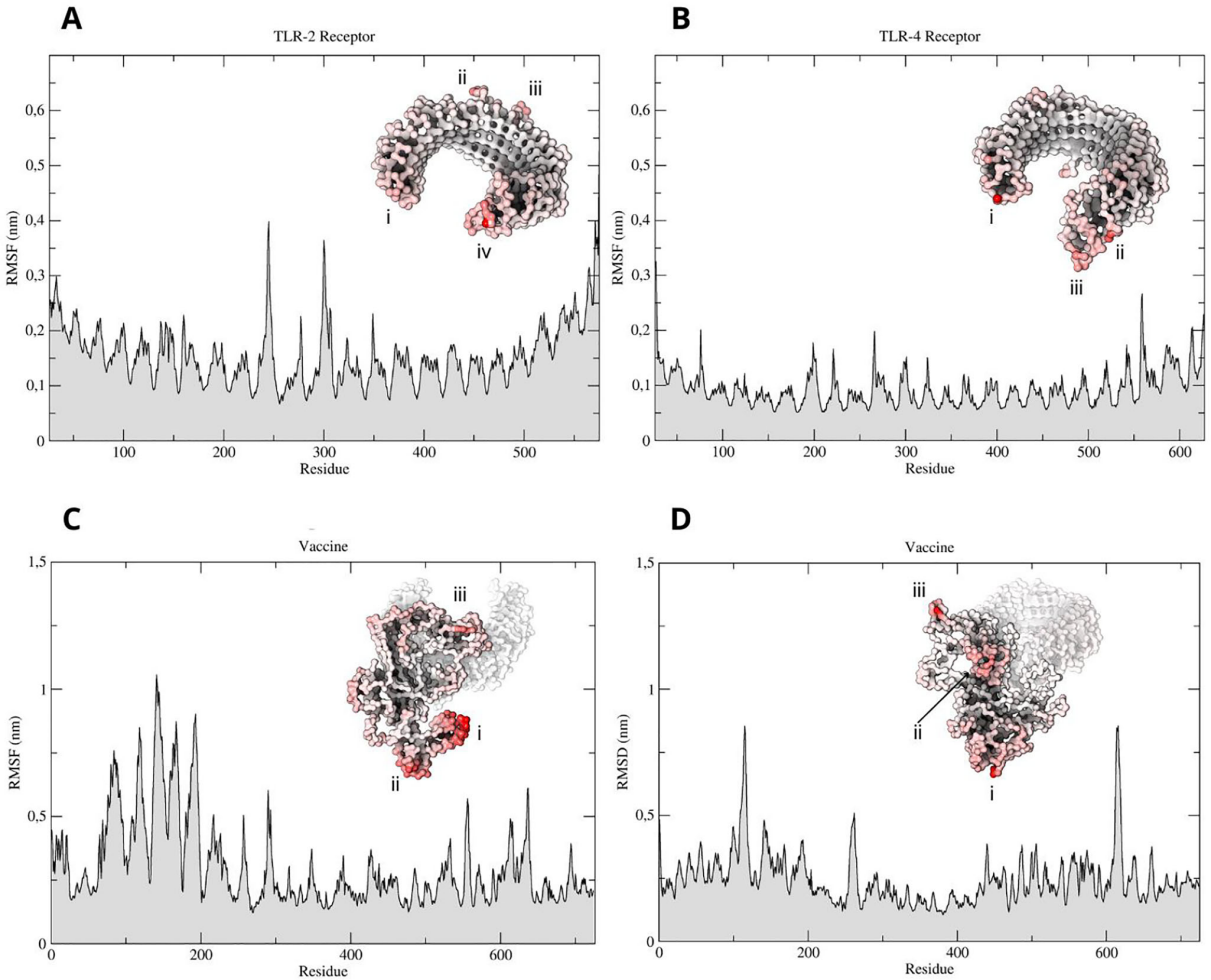
Root mean square fluctuation (RMSF) of the TLR2‐Vaccine and TLR4‐Vaccine complexes: (A) TLR2 receptor, (B) TLR4 receptor, (C) Vaccine in complex with TLR2 and (D) Vaccine in complex with TLR4. Surface cartoon represents the b‐factor structure. B‐factor representation highlighting the atomic displacement within the proteins. B‐factor coloring to indicate regions of varying flexibility. The red areas represent residues with higher B‐factors, signifying greater flexibility, while white areas represent more rigid regions of the protein.

Figure 8C,D present the RMSF values for the vaccine in complex with TLR2 and TLR4, respectively. The vaccine exhibits greater flexibility than the receptors, with RMSF values reaching up to 1.0 nm. In both systems, the vaccine's most flexible regions are found primarily in the surface and loop areas. Specifically, in the TLR2‐vaccine complex (Figure 8C), prominent peaks are observed around residues 80–250 (Region i and ii) and 550–650 (Region iii), highlighting flexible regions. Similarly, in the TLR4–vaccine complex (Figure 8D), superior flexibility is observed near residues 100–150 (Region i), 250–280 (Region ii), and 600–610 (Region iii). These regions of heightened flexibility are clearly represented by the intense red areas in the b‐factor surface illustrations, emphasizing the dynamic behavior of these segments. The high flexibility in these regions may facilitate receptor binding and adjustability, allowing the vaccine to interact more effectively with the receptors. Conversely, the binding pocket regions of the vaccine in both systems show reduced fluctuations, as indicated by lower RMSF values.

Structural and molecular flexibility analysis of the vaccine–TLR docked complex was executed by iMODs. Results from the iMODS tool evaluated the stability and physical movements of all the vaccine–TLR2 and –TLR4 docked complexes (Figures S3 and S4, respectively). The eigenvalues for the complexes were 1.35873 × 10^−5^ and 6.753441 × 10^−5^ for vaccine–TLR2 and –TLR4, respectively (Figures S3A and S4A), indicating the structural stability of the complexes. The plot visually demonstrates how the energy (in terms of eigenvalues) increases with the mode index. Higher eigenvalues generally correspond to motions with higher energy and lower frequency.

Figures S3B and S4B represent the percentage variance contributed by each of the first ten modes. Modes with higher variance percentages are more significant in defining the dynamics of the protein or molecular system. In both complexes, the variance increases with the mode index, showing the distribution of motion across different modes.

The covariance map of the docked complexes illustrated the correlated, uncorrelated, and anti‐correlated motions between a pair of residues using red, white, and blue colors, respectively (Figures S3C and S4C). Vaccine–TLR2 shows regions of correlated (red) and anti‐correlated (blue) motions, with several uncorrelated (white) areas, indicating complex interactions and functional flexibility. Vaccine–TLR4 displays extensive correlated and anti‐correlated regions, suggesting coordinated and opposing movements among residues, with significant anti‐correlated areas pointing to higher flexibility in certain parts of the complex.

Meanwhile, the elastic network model plot displays the connections between atoms and springs, where darker shades of grey signify greater rigidity in the springs (Figures S3D and S4D). Comparing both, vaccine–TLR4 appears to have more flexible areas than vaccine–TLR2, reflecting differences in their dynamic behavior and structural adaptability.

Similar to our results, a study by Khan et al. [85] identified a potential vaccine against *T. pallidum* using subtractive proteomics and reverse vaccinology approaches. MD simulation of the docked complexes with TLR receptors showed good stability, deformability, and low intrinsic value. Moreover, Soltan et al. [86] utilized a proteome‐based approach to identify candidates for a multiepitope vaccine against the Nipah virus. Their analysis of deformability and eigenvalues demonstrated favorable structural stability and functional adaptability for the resulting vaccine–receptor complex.

The MD simulations revealed key binding interactions within the vaccine–TLR2 and –TLR4 systems (Figures 9 and 10). The presence of hydrogen bonds, electrostatic, and also hydrophobic interactions confirms the stability and efficacy of vaccine candidates that interact with biological molecules such as TLR receptors [87, 88]. The vaccine–TLR2 complex shows important binding contacts, the hydrogen atom of ARG437 in TLR2 acts as a donor for the oxygen atom of the ligand GLU375, forming a hydrogen bond. Similarly, ARG534 binds to the GLU374. In addition, ARG321 in the ligand interacts electrostatically with ASP333 in the receptor. Another electrostatic interaction is observed between the ARG45 in TLR2 and GLU246 in the ligand. Moreover, ARG437 in TLR2 interacts electrostatically with the GLU375 in the ligand. Similarly, ARG488 and ARG534 in TLR2 interact with the GLU375 in the ligand.

**FIGURE 9:**
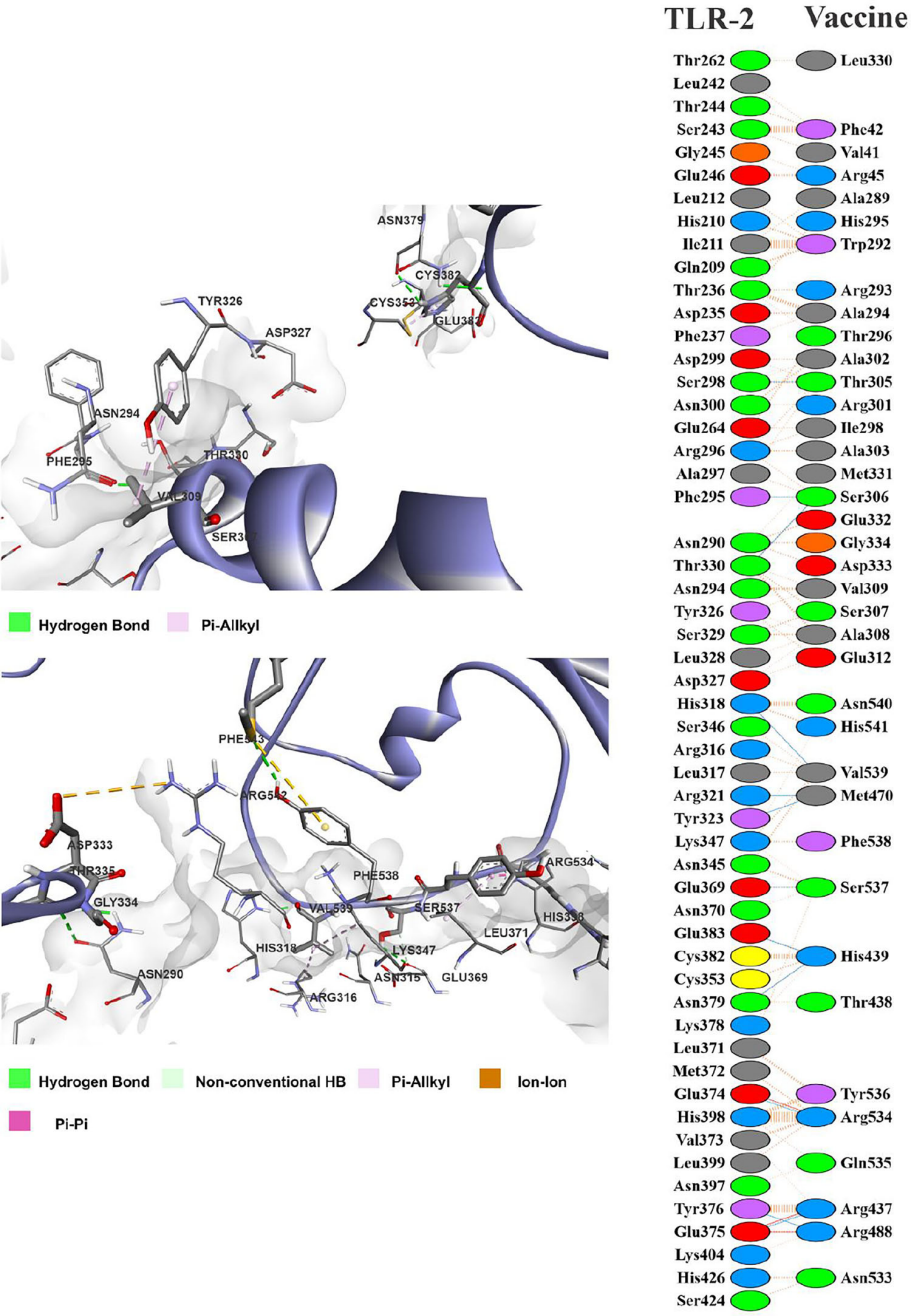
The figure illustrates the molecular interactions between TLR‐2 and a vaccine. Two panels show different interaction pockets of the TLR‐2 structure that interact with the vaccine at specific residues. The right panel maps all interacting residues and bonds between the vaccine and TLR‐2 protein chains.

**FIGURE 10:**
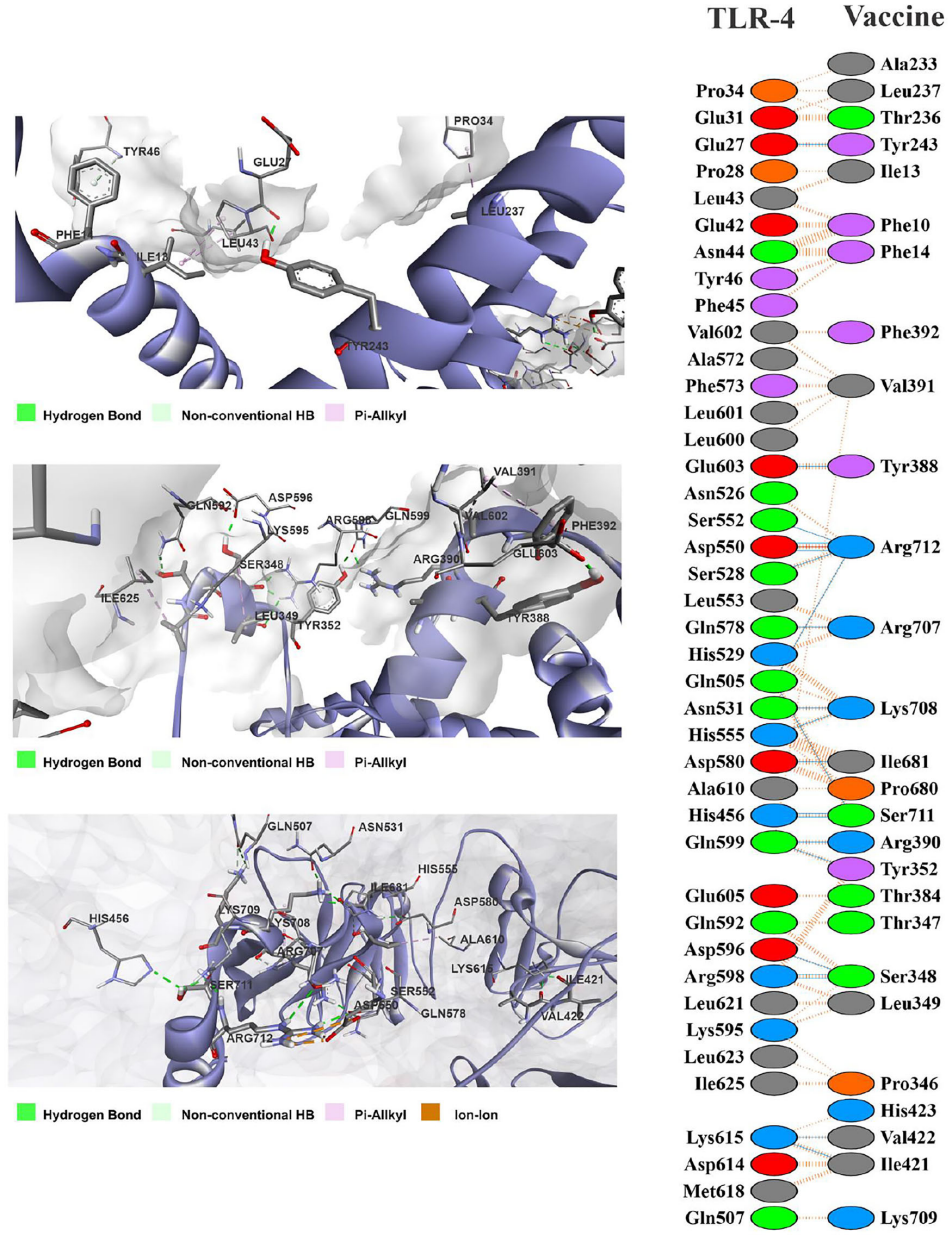
The figure illustrates the molecular interactions between TLR‐4 and a vaccine. Three panels show different interaction pockets of the TLR‐4 structure that interact with the vaccine at specific residues. The right panel maps all interacting residues and bonds between the vaccine and TLR‐4 protein chains.

Conventional hydrogen bonds are also formed between ASN290 in the ligand and GLU332 in TLR2, and HIS318 in the ligand and VAL539 in TLR2. TYR323 interacts with MET470 in TLR2. Similarly, TYR376 in the ligand forms a hydrogen bond with the oxygen atom of ARG488 in TLR2. Further, ASN379 (ligand) interacts with the THR438 (receptor), ASN290 with GLY334 and ASN379 with HIS439, forming hydrogen bonds. In addition, GLU369 forms a bond with SER537.

Carbon–hydrogen bonds are also observed between THR236 and THR296, and between GLU369 and SER537. TYR323 (ligand) interacts with MET470 (receptor), forming a pi–sulfur interaction. In addition, HIS398 interacts with TYR536 via pi–pi stacking. HIS210 forms a pi–pi with TRP292. Further hydrophobic interactions include ARG316 with VAL539 and LYS347 with VAL539. The HIS210 also forms a pi–alkyl interaction with ALA294 and HIS238 interacts with ALA285. Similarly, TYR326 forms a pi–alkyl interaction with VAL309. Finally, CYS353 (ligand) forms a pi–alkyl interaction with HIS439 (receptor) and LEU371 interacts with TYR536.

The vaccine–TLR4 complex shows significant binding contacts. The ASP550 (ligand) forms a conventional hydrogen bond with ARG712 (receptor). In another interaction, ASP550 binds with ARG712. In addition, ASP550 interacts electrostatically with ARG712. The GLN592 binds THR347. Similarly, ARG598 interacts with SER348, forming bonds. In addition, the ARG598 binds with LEU349. The LYS615 forms hydrogen bonds with ILE421 and VAL422. GLU27 binds with TYR243 and ASP596 with SER348.

Moreover, GLN599 interacts with TYR352, forming a bond. GLU603 binds with TYR388. In addition, GLN599 interacts with ARG390, forming a bond, such as ASP580 with ILE681. GLN578 interacts with ARG707 to form a conventional hydrogen bond and, similarly, HIS555 interacts with the LYS708. Finally, GLN505 binds with SER711, and GLN505 interacts with ARG712.

### 3.12 Codon Optimization of the Designed Vaccine Peptide for Expression Analysis

To express the chimeric protein in an expression system, the amino acid sequence was reverse translated by the backtranseq program of EMBOSS 6.0.1. The choice of the *E. coli* expression system during the in silico cloning process is essential for effective vaccine production. The nucleotide sequence of the vaccine was used as input in the Java codon adaptation program (JCat) to adapt the codons to the prokaryotic organisms that are mostly sequenced. The Java codon adaptation tool was used to perform codon optimization. The optimized codon sequence, consisting of 2150 nucleotides, initially had a GC content of 56.76%. After undergoing optimization with the JCat, the GC content was adjusted to 49.30%, closely aligning with the GC content of strain K12, which is approximately 50.73%. In addition, before codon adaptation, the Codon Adaptation Index (CAI) of the sequence was 0.56; post‐optimization, the CAI improved to 1. A CAI above 0.8 and a GC content between 30% and 70% indicate good protein expression in the host system [54]. Using SnapGene software, the optimized sequence was cloned into the pET‐28a (+) vector. Eco53kI and BstZ17I restriction sites were incorporated to create flanking sites at the ends of both the construct and the plasmid, facilitating the expression of the vaccine construct (Figure 11).

**FIGURE 11:**
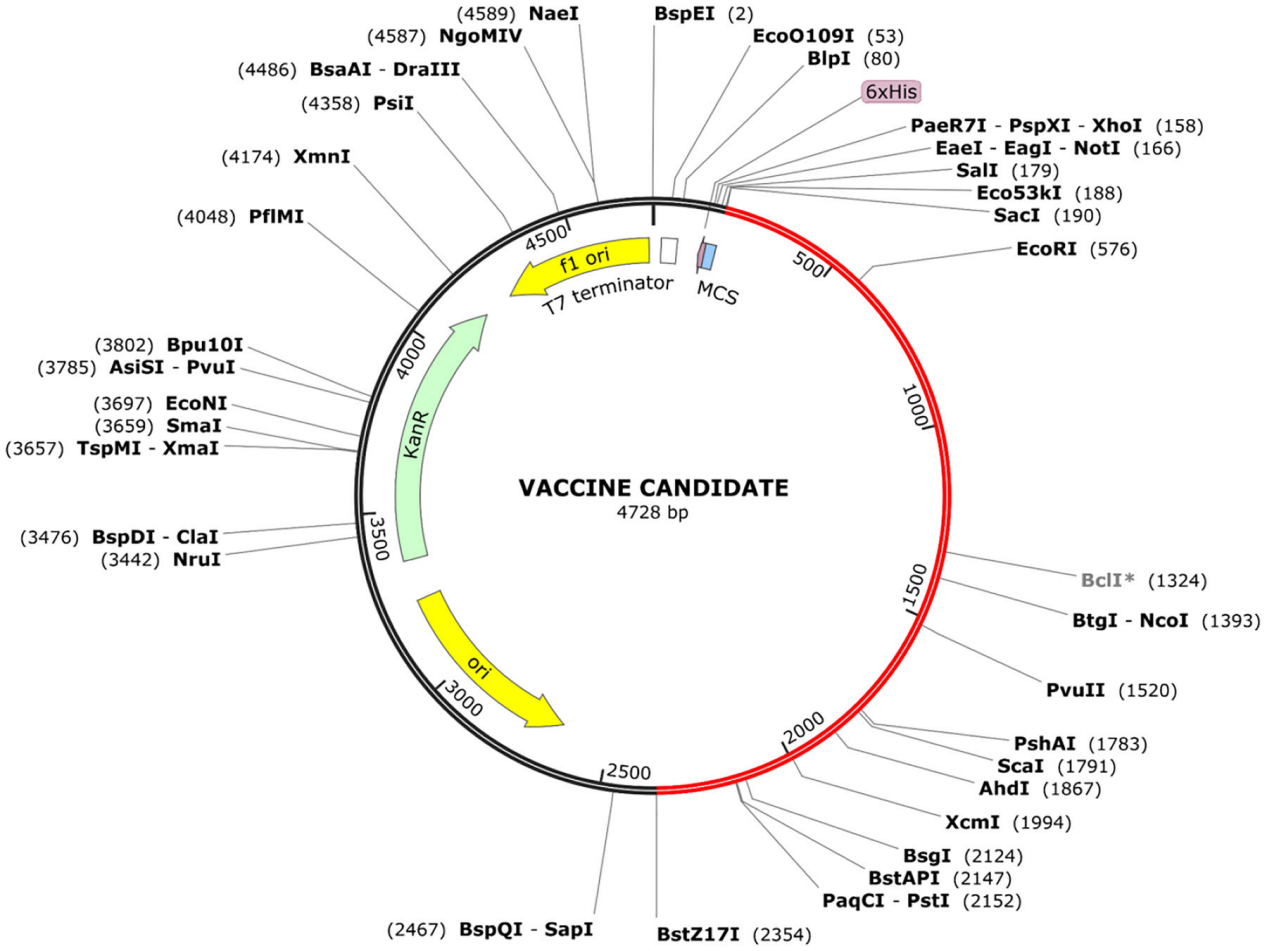
In silico cloning of the vaccine construct. The pET‐28 (+) backbone is shown in black and the vaccine construct is shown in red, surrounded between EC53KI (188) and BstZ1ZI (2354).

### 3.13 Immune Response Simulation

The C‐ImmSim server enables the evaluation of candidate vaccine immunogenicity through computer simulations, facilitating the rapid screening of potential vaccines. The C‐ImmSim model effectively illustrates the innate, humoral, and cell‐mediated immune responses to antigens. As an intracellular pathogen, *T. cruzi* requires both innate and, primarily, cellular and humoral immunity for effective clearance and destruction. Our immune mimicry predictions demonstrated that the Chagas vaccine candidate can efficiently prime the innate immune system to induce robust immune responses. Specifically, we observed the proliferation of macrophages and dendritic cells (DC) following vaccine stimulation (Figure 12).

**FIGURE 12:**
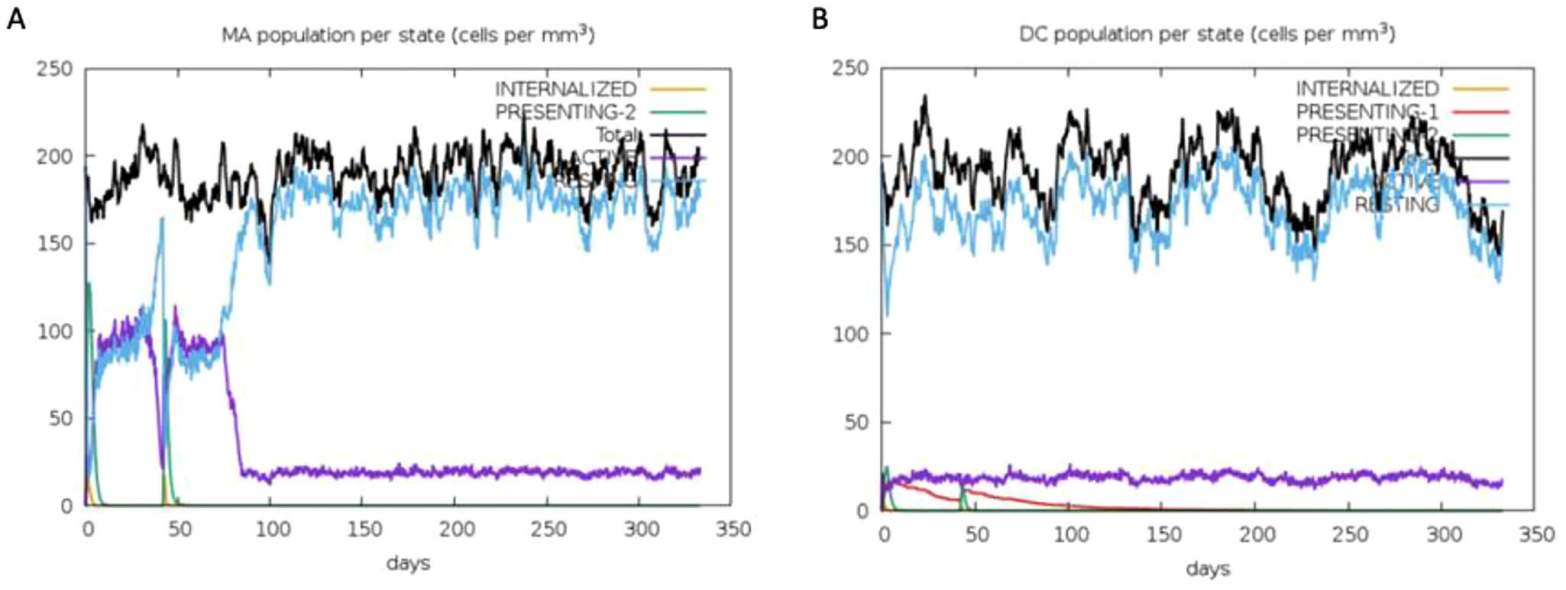
Innate immune response induced by Chagas vaccine prototype in the C‐ImmSim servers. (A) Active macrophages (purple) following stimulation with vaccine; (B) DC levels (purple) following vaccine stimulation.

The C‐ImmSim server predicted that following vaccine stimulation, B lymphocytes primarily perform antigen‐presenting functions during the early phase (Figure 13A). The active B cell population per condition then proliferated substantially, maintaining elevated levels even 300 days after vaccine stimulation (Figure 13A). Notably, the population of memory B cells increased (Figure 13B), along with the production of IgM and IgG antibodies after vaccine administration (Figure 13C). Following the second dose, there was a substantial increase in the production of these immunoglobulins, particularly IgG1, an immunoglobulin known to be a potent trigger of effector mechanisms crucial for parasite elimination [89].

**FIGURE 13:**
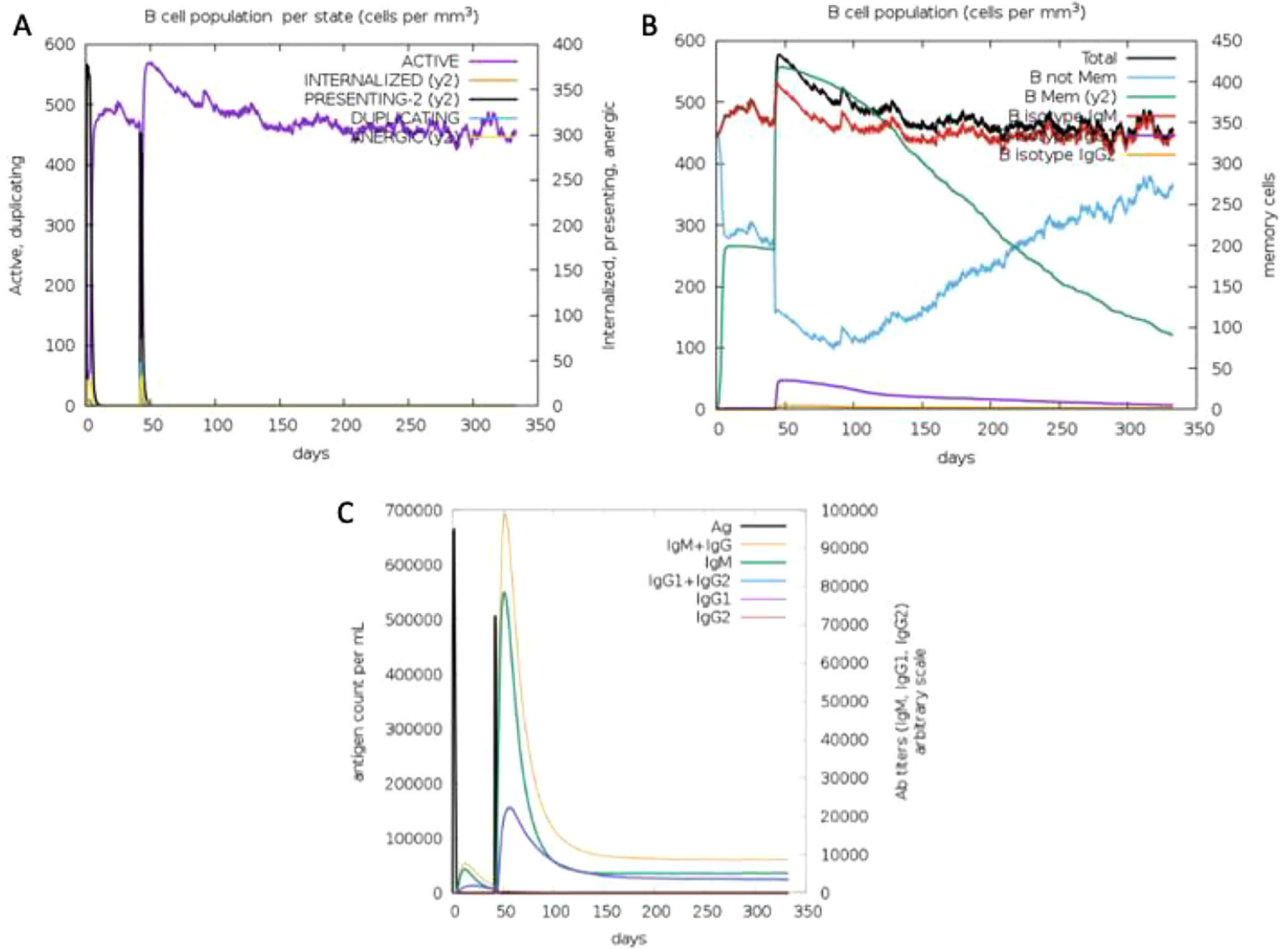
Humoral immune response induced by Chagas vaccine prototype in the C‐ImmSim servers. (A) Secretion of active B‐cells (purple) following antigenic stimulation; (B) Production of memory B‐cells (green) after antigenic stimulation; (C) Immunoglobulin production from antigen injection (black vertical line); colored peaks denote immunoglobulin subclasses.

A coordinated response between Th1 and Th2 profiles is desirable, with a predominance of Th1 effector mechanisms for the control and elimination of the parasite [90]. The data indicate an increased and durable production of active T helper cells after the administration of the second dose (Figure 14A), with this population being predominantly of the Th1 type (Figure 14B). In addition, the vaccine‐induced T helper memory cells for more than 300 days (Figure 14C).

**FIGURE 14:**
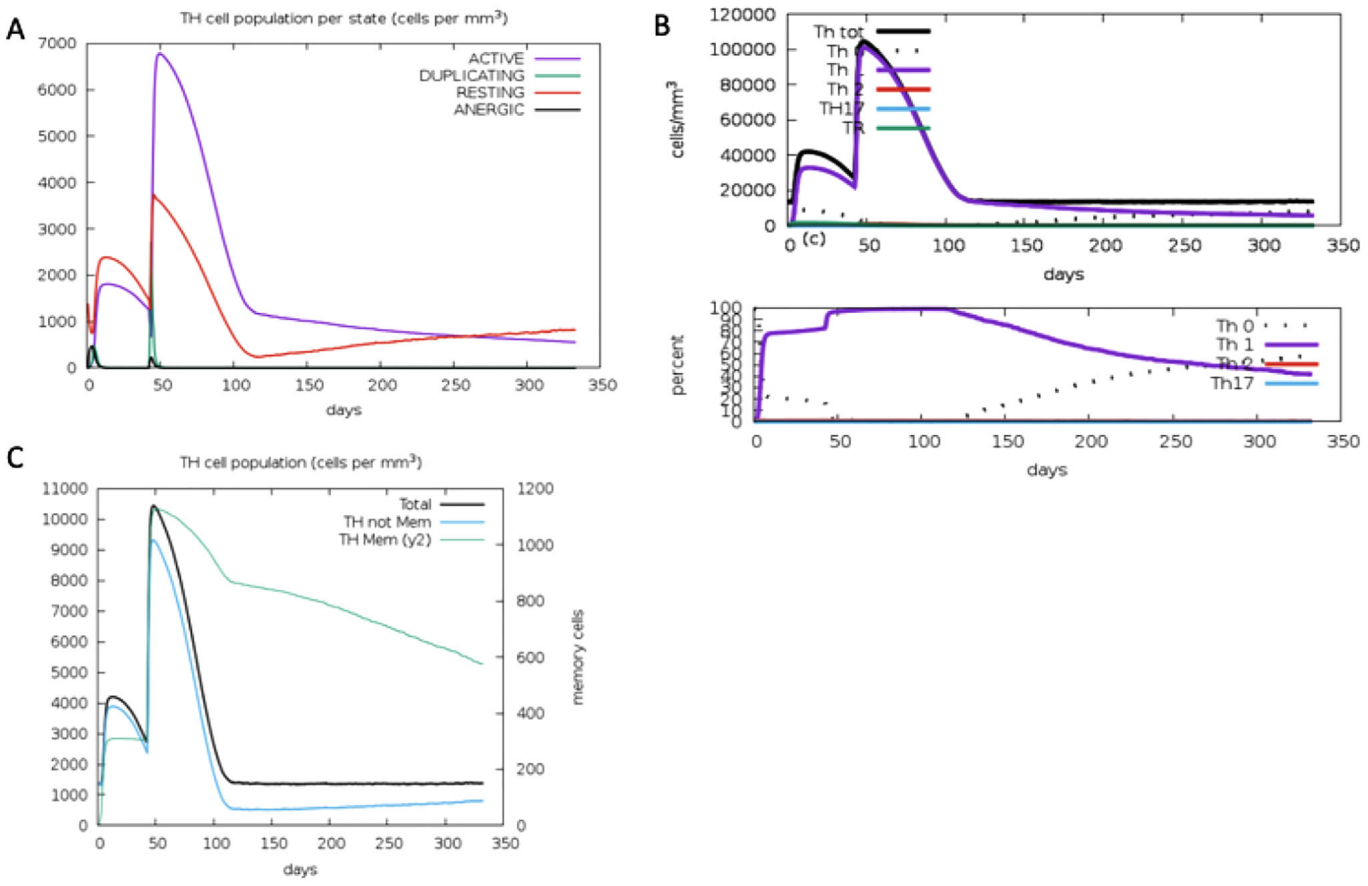
Cellular immune response induced by Chagas vaccine prototype in the C‐ImmSim servers. (A) Increased production of active T helper cells after vaccine doses (purple) (CD4 T‐helper lymphocytes count sub‐divided per entity‐state—i.e., active, resting, anergic and duplicating); (B) The vaccine prototype induce Th1 profile in T helper cells after stimulation (purple); (C) The vaccine stimulation is capable to induce memory T helper cells for long time (green) (CD4 T‐helper lymphocytes count. The plot shows total and memory counts).

We also found that the number of active cytotoxic T cells gradually increased, peaking around day 30 after stimulation (Figure 15A). The activation of CD8 T cells results in the acquisition of the molecular machinery necessary for the elimination of target cells, which includes cytoplasmic granules containing proteins such as perforin and granzymes, as well as the production of cytokines like IFN‐γ and IL‐2 [91]. Upon vaccine administration, an increase in pro‐inflammatory cytokines such as IFN‐γ and IL‐2 was observed, which are essential for promoting an adequate immune response to eliminate *T. cruzi* (Figure 15B).

**FIGURE 15:**
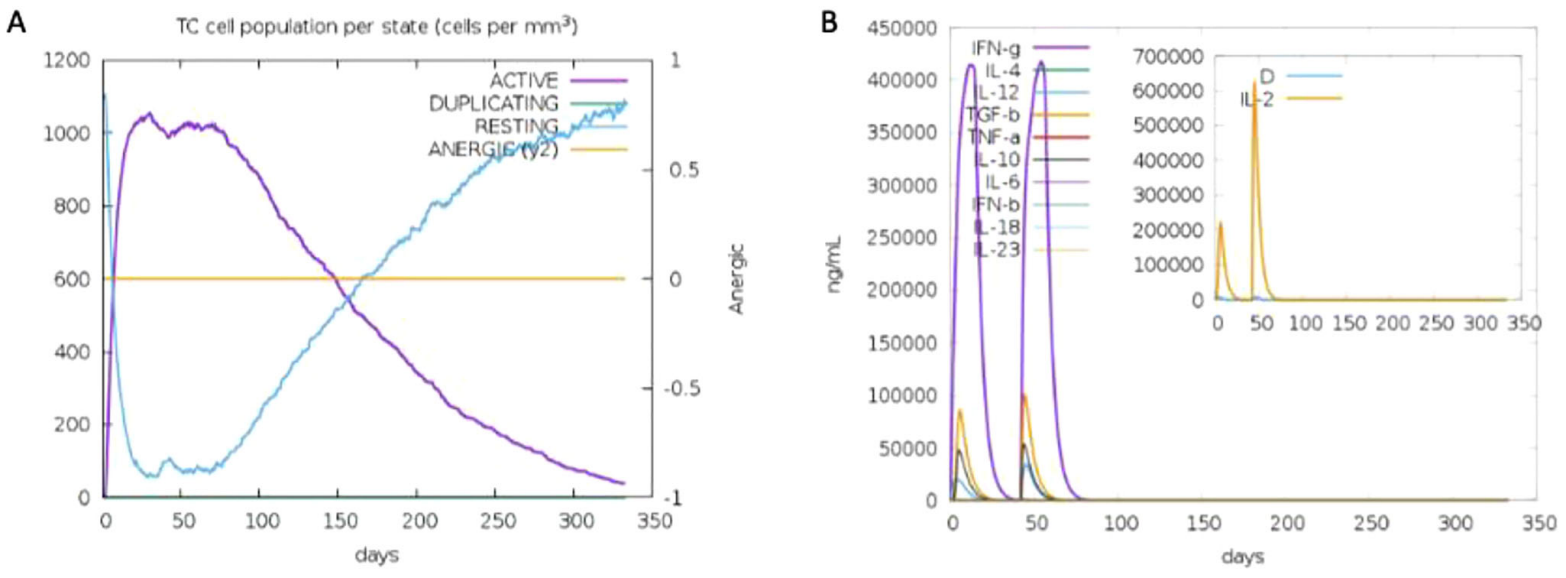
(A) CD8 T‐cytotoxic lymphocytes count per entity‐state. (B) Concentration of cytokines and interleukins. Inset plot shows danger signal together with leukocyte growth factor IL‐2.

## 4 Conclusion

Our study focused on the development of vaccine candidates against Chagas disease, utilizing the proteins ASP‐2 and gp82 from the *T. cruzi* parasite. We identified epitopes of ASP‐2 and gp82 characterized by high antigenicity, immunogenicity, non‐toxicity, and broad population coverage, indicating their potential to elicit a robust immune response.

A chimeric vaccine was meticulously constructed, incorporating multiple T‐cell and B‐cell epitopes linked with adjuvants and linkers, demonstrating favorable physicochemical properties and structural stability. Molecular docking and dynamic molecular simulations revealed significant interactions between the vaccine construct and immune receptors TLR2 and TLR4, which are vital for modulating immune responses. The population coverage analysis indicated that the selected epitopes effectively cover a significant portion of the Latin American population, which is crucial for ensuring the vaccine's efficacy across diverse epidemiological settings.

Immune response simulations showed that the vaccine could induce robust innate and adaptive immune responses, marked by the production of antibodies and the activation of helper and cytotoxic T‐cells, essential for the effective clearance of *T. cruzi*. These findings suggest that the developed vaccine holds significant potential to fight against *T. cruzi* infection, providing a solid foundation for future studies and clinical development.

## Author Contributions

**João Firmino Rodrigues‐Neto**: conceptualization, methodology, software, formal analysis, writing – original draft, writing – review and editing, supervision. **Maria Karolaynne da Silva**: methodology, investigation, data curation. **Caio Patrício de Souza Sena**: methodology, software, investigation. **Davi Serradella Vieira**: methodology, investigation. **Magdi E. A. Zaki**: writing – review and editing. **Leow Chiuan Yee**: writing – review and editing. **Shahina Akter**: writing – review and editing. **Umberto Laino Fulco**: formal analysis, writing – review and editing. **Jonas Ivan Nobre Oliveira**: conceptualization, formal analysis, writing – review and editing. All authors have read and agreed to the published version of the manuscript.

## Conflicts of Interest

The authors declare no conflicts of interest.

## Supporting information




**Supplementary Tables**: bab70185‐sup‐0001‐Tables.xlsx

**Supplementary Figures**: bab70185‐sup‐0002‐Figures.docx

## Data Availability

Data will be made available on request.
