# Supplementary material for: Targeting Amastigote and Trypomastigote Phases: Multi‐Epitope Vaccine Strategy Against Trypanosoma cruzi
Source: Biotechnol Appl Biochem. 2026 May 29;73(5):2457–81. doi: 10.1002/bab.70185 (PMC13644382; doi:10.1002/bab.70185)
Supplement: Supplementary file 1 [file BAB-73-2457-s001.docx]

Supplementary Figure S1. Displayed are the ERRAT analyses graphs for a vaccine model, assessing structural quality (A) before and after refinement; and (B) Verify 3D focusing on the compatibility evaluation of protein 3D structures before and after refinement.

Supplementary Figure S2. This graph is generated by ProSAweb for vaccine showing the quality of 3D model on the basis of z-score for vaccine construct before and after refinement.

Supplementary Figure S3. iMODs simulation of Vaccine-TLR2 docked complex. (A) The eigenvalue showing the energy required to deform the structure; (B) The variance associated to each normal mode is inversely related to the eigenvalue. Colored bars show the individual (purple) and cumulative (green) variances; (C) Covariance matrix indicates coupling between pairs of residues; and (D) The elastic network model, suggesting the connection between atoms and springs.

Supplementary Figure S4. iMODs simulation of Vaccine-TLR4 docked complex. (A) The eigenvalue showing the energy required to deform the structure; (B) The variance associated to each normal mode is inversely related to the eigenvalue. Colored bars show the individual (purple) and cumulative (green) variances; (C) Covariance matrix indicates coupling between pairs of residues; and (D) The elastic network model, suggesting the connection between atoms and springs.
